# Sexual Dysregulation After Traumatic Brain Injury and Stroke: A Critical Narrative Review of Neurobiology, Clinical Phenotypes, and Management

**DOI:** 10.3390/brainsci16090995

**Published:** 2026-09-19

**Authors:** Rocco Salvatore Calabrò, Rosaria De Luca, Riccardo Raul Ruberto, Andrea Calderone, Demetrio Milardi, Francesco Tomaiuolo

**Affiliations:** 1 Department of Neurorehabilitation, IRCCS Centro Neurolesi Bonino Pulejo, 98124 Messina, Italy; roccos.calabro@irccsme.it (R.S.C.); rosaria.deluca@irccsme.it (R.D.L.); riccardo.ruberto@irccsme.it (R.R.R.); 2 Department of Biomedical, Dental Sciences and Morphofunctional Imaging, University of Messina, 98125 Messina, Italy; demetrio.milardi@unime.it; 3 Department of Clinical and Experimental Medicine, University of Messina, 98125 Messina, Italy; francesco.tomaiuolo@unime.it

**Keywords:** acquired brain injury, traumatic brain injury, stroke, hypersexuality, sexual disinhibition, acquired paraphilic manifestations, orbitofrontal cortex, neurorehabilitation

## Abstract

**Highlights:**

**What are the main findings?**

- Human sexual regulation emerges from the coordinated, context-sensitive interaction of executive-control, salience, reward, interoceptive, memory, endocrine–autonomic, and social–cognitive systems; no single brain region constitutes a deterministic “sexual center.”
- Traumatic brain injury and stroke provide complementary mechanistic models: TBI highlights diffuse or multifocal regulatory-network vulnerability, whereas stroke can nominate strategic lesion-connected nodes. In both conditions, clinical expression remains heterogeneous, probabilistic, and context-dependent.

**What are the implications of the main findings?**

- Assessment should begin with precise behavioral phenotyping and differential formulation, followed by evaluation of decision-specific capacity, consent, collateral information, safeguarding, and interdisciplinary neurorehabilitation.
- Management should use the least restrictive targeted strategy, with explicit monitoring of benefit and harm and predefined de-escalation criteria. Future studies should combine standardized phenotypes, prospective multicenter cohorts, lesion-network and connectivity methods, and neuropsychological and ecological outcomes.

**Abstract:**

**Background/Objectives:** Acquired brain injury (ABI) can disturb sexual regulation, but the literature does not support a single lesion-to-behavior relationship. This critical narrative review evaluates hypersexuality, sexual disinhibition, and rare acquired paraphilic manifestations after traumatic brain injury (TBI) and stroke, and asks how sparse, heterogeneous evidence can inform proportionate clinical care. **Methods:** PubMed/MEDLINE, Scopus, and Web of Science were searched up to 6 August 2026. Two reviewers independently screened records and full texts. Evidence appraisal considered methodological quality, directness, and competing explanations. Thirteen English-language case-based publications were assessed, with neurological observations distinguished from pharmacological comparators. **Results:** Sexual dysfunction is common after ABI, whereas dysregulated sexual behavior is less frequent and poorly quantified. TBI primarily supports a distributed vulnerability model involving diffuse axonal injury and disruption of frontal regulatory connections. Stroke can nominate strategic candidate nodes, but lesion-network effects and clinical context remain decisive. Clinical observations associate inappropriate sexual behavior with broader behavioral difficulties, without establishing a uniform syndrome. Recent guidelines support individualized sexuality care, while contemporary imaging studies strengthen network plausibility without directly establishing sexual-dysregulation mechanisms. **Conclusions:** A phenotype-first framework separates dysfunction from dysregulation and distinguishes direct clinical evidence from mechanistic extrapolation. Assessment should establish what changed and whether reversible contributors explain the behavior before assigning a persistent phenotype. Management should match the dominant clinical problem, preserve consensual intimacy, and use the least restrictive targeted strategy. Explicit monitoring should determine whether benefit justifies continued treatment. Case-based evidence and language-restricted selection limit generalizability; prospective studies require standardized phenotypes and repeated observation across settings.

## 1. Introduction

Sexuality combines subjective desire and bodily arousal with the relational meaning of intimacy. Its expression depends on memory, social understanding, and the ability to regulate behavior. A clinically useful distinction is therefore required between sexual function—desire, arousal, orgasm, and activity—and sexual regulation: the capacity to use consent, privacy, interpersonal boundaries, and anticipated consequences to guide expression. Classical lesion-based neuropsychology linked ventromedial prefrontal injury to impaired real-life decision-making despite relatively preserved conventional cognitive performance [1,2]. Functional neuroimaging of sexual arousal and reward subsequently implicated interacting prefrontal, cingulate, limbic, striatal, hypothalamic, insular, sensorimotor, and autonomic systems [3,4,5]. Contemporary post-TBI functional MRI has further demonstrated treatment-related modulation of corticostriatal connectivity associated with executive function [6]. These complementary findings favor distributed regulation over a unitary ‘sexual center’, but executive-function and reward-task findings provide mechanistic support rather than direct validation of post-ABI sexual dysregulation. Earlier erotic-stimulus and orgasm studies provide additional physiological context [7,8,9,10].

After acquired brain injury (ABI), reduced desire, erectile or orgasmic difficulty, fatigue, altered body image, and relationship disruption are more common than dysregulated sexual behavior [11,12]. A 2025 meta-analysis of 279 prospective stroke cohorts (117,440 participants) estimated a pooled sexual-dysfunction prevalence of 59.8%, although heterogeneity was high and the included outcomes did not quantify hypersexuality or disinhibition [13]. A smaller but clinically important group develops hypersexuality, sexual disinhibition, compulsive or perseverative behavior, context-inappropriate comments or contact, risk-taking, or rare acquired paraphilic manifestations [14,15,16,17]. These presentations can disrupt rehabilitation, expose patients or others to harm, and generate stigmatizing or legally consequential interpretations.

The phenotypes overlap but are not interchangeable. Hypersexuality concerns increased desire or preoccupation; sexual disinhibition concerns difficulty controlling expression in context. Intrusive behavior can therefore occur without stronger desire. Compulsive sexual behavior emphasizes repetition that is difficult to control, while context-inappropriate behavior describes an observable mismatch without prescribing its mechanism. Rare ‘acquired paraphilic manifestations’ require separate consideration of interest, enacted behavior, and disorder; none establishes a deterministic lesion effect [18,19,20]. Sexual dysfunction describes impaired or unsatisfactory sexual function, whereas dysregulation describes difficulty governing expression in relation to consent, privacy, boundaries, or consequences; they can coexist. At the bedside, subjective desire and physiological function should therefore be recorded separately from observed behavior. A consistently defined observation log should capture the antecedent, exact behavior, setting, opportunity for observation, response to redirection, and consequences, without interpreting frequency alone as pathology. Increased consensual activity with preserved control differs from intrusive contact despite unchanged desire. Section 7.1 translates this distinction into a structured clinical assessment.

Much of the dysregulation literature consists of selected cases, small series, retrospective observations, and indirect models. Recent guidelines and rehabilitation studies address sexuality support after TBI, but do not establish the incidence, neuroanatomy, or treatment efficacy of rare dysregulated phenotypes [21,22]. Incomplete premorbid characterization makes it difficult to determine what changed after injury. Unrecorded medication exposure or changes in supervision further weaken interpretation of the temporal association.

The rationale for this review is therefore not to propose another ‘sexual center’, but to close a translation gap between three literature bases that are usually discussed separately: broad post-ABI sexual dysfunction and service needs; rare reports of dysregulated or paraphilic manifestations; and domain-general evidence on executive, salience, reward, awareness, and lesion-connected networks. Without this integration, common dysfunction can be confused with rare dysregulation, striking lesion reports can be overlocalized, and management can become either undertreated or unnecessarily restrictive.

TBI and stroke offer complementary rather than competing inferences. TBI commonly combines diffuse axonal injury, frontotemporal contusions, and frontostriatal or frontolimbic disconnection, making it most informative about distributed regulatory vulnerability. Stroke can provide clearer temporality and strategically located lesions, but focal anatomy remains embedded within connected networks and clinical context. Neither etiology supports a one-lesion-one-behavior model.

Sexuality also remains under-assessed in neurological care because patients, partners, and clinicians may avoid discussion owing to shame, discomfort, limited training, communication impairment, or uncertainty about consent and legal risk. The review first defines clinically separable phenotypes, then compares what TBI and stroke can validly contribute to causal inference. This comparison constrains neuroanatomical claims and distinguishes direct ABI evidence from mechanistic or non-ABI extrapolation. It provides the basis for an evidence-graded assessment and management framework. The aim is a clinically usable map of uncertainty.

### Methods

We conducted a structured critical narrative review guided by recommendations for transparent narrative synthesis and the Scale for the Assessment of Narrative Review Articles (SANRA) [23,24,25]. The six SANRA domains address rationale, aims, search transparency, referencing, scientific reasoning, and presentation of relevant endpoint data. SANRA was used to guide reporting and final internal quality appraisal, not as a substitute for a systematic-review protocol or as a numerical claim of certainty.

Two authors (R.S.C. and A.C.) searched PubMed/MEDLINE, Scopus, and Web of Science from database inception through 6 August 2026, restricted to English-language publications. Search blocks combined ABI etiology terms with phenotype terms and mechanism, anatomy, assessment, or management terms. The same two authors collated records, removed duplicates, and conducted backward and forward citation checking for landmark clinical and methodological sources. Exact source-specific strings, fields, coverage, and dates are reported in Supplementary Table S1.

Two reviewers (R.S.C. and A.C.) independently screened titles and abstracts. Records considered potentially relevant by either reviewer underwent independent full-text assessment by both; disagreements were resolved through discussion and consensus. The case-based synthesis comprised 13 English-language publications available in full text. Case reports without an available full text were excluded from this synthesis. Supplementary Table S2 reports their bibliographic and clinical characteristics; Supplementary Table S3 details treatment, follow-up, outcomes, and limitations. Direct TBI/stroke observations are distinguished from neurological and pharmacological comparators. Publication counts were not pooled as patient counts. The 13-publication register comprises ten neurologically informative case reports or case series and three non-ABI pharmacological comparators. The ten neurological publications are Miller [14], Britton [15], Spinella [17], Alnemari [19], Burns and Swerdlow [20], Eghwrudjakpor and Essien [26], Lilly [27], Caro and Jimenez [28], Mutarelli [29], and Sartori [30]. They include direct TBI/stroke observations, mixed-etiology series, and neurological comparators; they are neither ten homogeneous single-case reports nor ten independent lesion-localization experiments. The remaining publications are the sertraline-associated hypomania report [31] and two non-ABI naltrexone treatment reports [32,33]; their comparator roles are explicit in Supplementary Tables S2 and S3.

Eligible evidence included clinical studies, rehabilitation cohorts, case reports or series, lesion observations, neuroimaging studies, mechanistic reviews, clinical guidelines, implementation studies, and management papers that directly informed post-ABI sexual dysregulation or a necessary differential mechanism. We excluded duplicate publications, non-English reports, papers without relevant ABI, phenotype, mechanism, assessment, or management content, and non-ABI sources that did not provide an explicitly necessary mechanistic or clinical comparator. Direct sexual-dysregulation evidence was distinguished from broader sexual-function or service evidence and from domain-general mechanistic evidence. Recent studies were preferred for equivalent claims; older publications were retained only when foundational, instrument-validating, or the only direct clinical evidence for a specific presentation.

The case-based synthesis was qualitative. The publication register documents the assessed reports but does not establish exhaustive retrieval or support pooled estimates of prevalence or treatment effects. Eligibility was restricted to English-language full texts to permit direct appraisal of clinical details. This restriction may omit relevant evidence and introduce language and cultural bias.

Two authors (R.S.C. and A.C.) undertook charting, critical appraisal, treatment-evidence grading, and synthesis. Charting connected study design and population to phenotype, anatomy, and clinical course. Appraisal examined premorbid characterization, follow-up, and competing explanations, alongside sex and gender reporting and the source of behavioral observations; Supplementary Table S1 specifies the fields. Case reports and series remained hypothesis-generating. Treatment evidence was graded using the 2011 Oxford Centre for Evidence-Based Medicine hierarchy, distinguishing direct post-ABI evidence from extrapolation [34].

For the qualitative case synthesis, retention was based on an identifiable phenotype or relevant differential syndrome, a defined neurological or pharmacological context, and a contribution to timing, anatomy, assessment, or management. Anatomical detail was not treated as a requirement for causal credibility: demonstrated lesions were distinguished from clinically inferred localization, and reports with missing imaging or premorbid data were retained only with those limitations explicit. Cases were compared by phenotype, etiology, temporal course, premorbid characterization, competing explanations, and recovery or treatment sequence, then interpreted against cohort observations and domain-general evidence. Contradictory courses and normal test findings were preserved. SANRA assessed narrative reporting, not the validity of individual cases, and no SANRA cutoff was used to select the ten neurological publications.

Publication and reporting bias were handled through claim-specific qualitative appraisal rather than a statistical correction. Incomplete premorbid sexual or psychiatric histories were treated as unreported, not as negative histories; retrospective collateral accounts were distinguished from independently documented baselines. An uncertain baseline limited inference to newly observed behavior and did not establish a newly acquired preference. Medication exposure, mood, supervision, opportunity, follow-up, and possible duplicate reporting further constrained causal interpretation. Neither the frequency of published cases nor the apparent recurrence of an anatomical location was used as an incidence estimate or a measure of localization certainty. Funnel-plot methods were not applicable to this non-comparative, non-exhaustive case register. Supplementary Table S1 makes these appraisal rules explicit.

The conceptual figures were developed from the evidence synthesis and established neurobehavioral literature [35]. OpenNeuro dataset ds006072, version 1.1.0 [36], was used solely to provide de-identified anatomical T1-weighted MRI backgrounds; no participant-level, functional, medication, or outcome data from the dataset were analyzed. Colored overlays are author-controlled schematic annotations, not atlas-derived or subject-specific segmentations, and do not imply lesion-behavior causality.

Focused reference checking on 14 and 17 September 2026 supplemented the database searches with literature on foundational lesion evidence, dopaminergic agents, stroke rehabilitation, research ethics, and endocrine contributors. These sources informed the interpretative framework and were appraised separately from the 13-publication case-based synthesis.

## 2. Functional Neurobiology of Sexual Regulation

### 2.1. Distributed Functional Systems

Sexual regulation is an emergent function of interacting systems that generate arousal and incentive value, place cues within autobiographical and relational context, represent bodily state, detect social signals, and inhibit or redirect behavior. This distinction is more informative for ABI than a search for a single sexual center because inappropriate behavior may occur despite unchanged or reduced desire. The functional-anatomical evidence and its limits are summarized in Supplementary Table S4 and are consistent with broader clinical synthesis [35].

**Figure 1:**
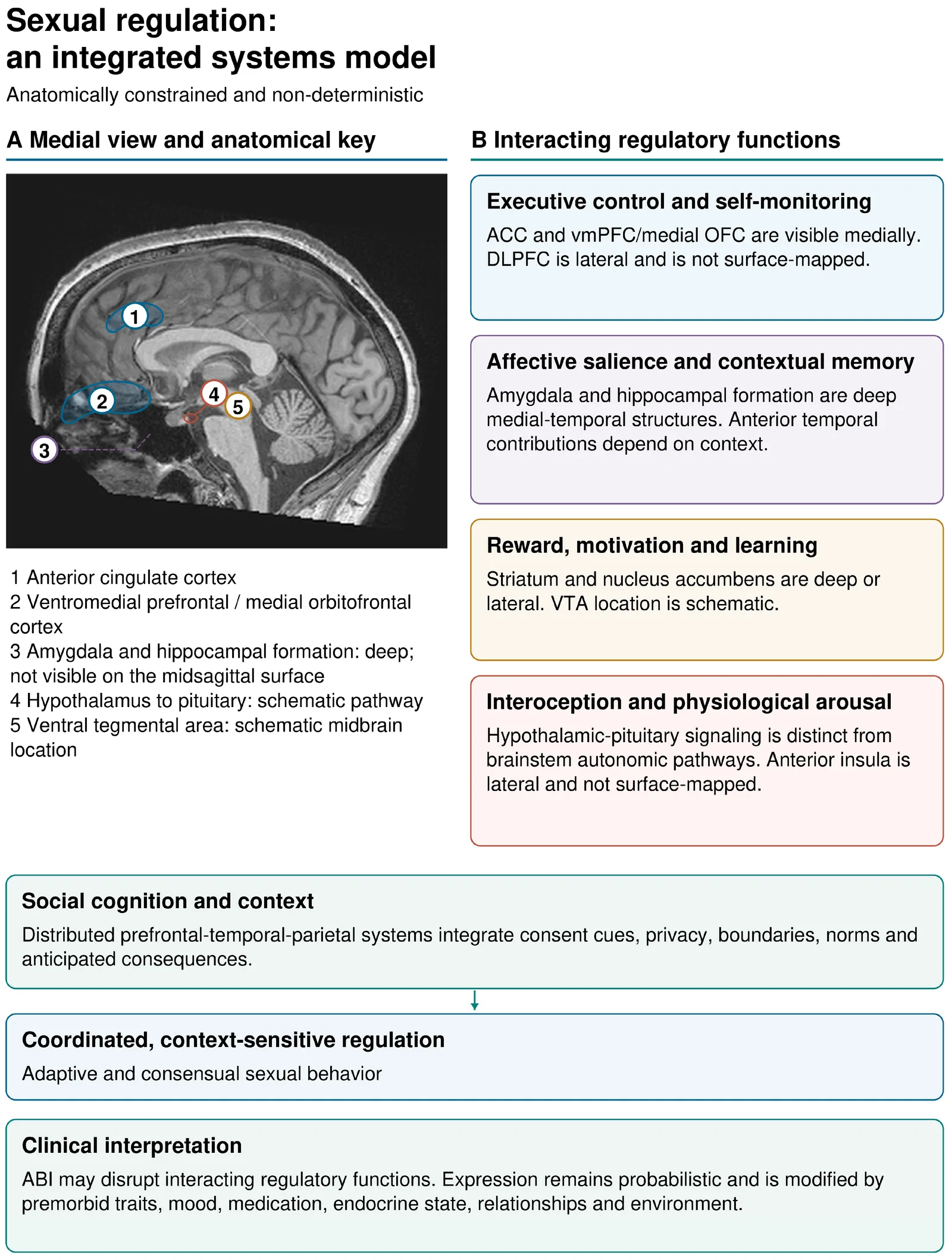
Integrated functional-neuroanatomical model of sexual regulation. A left midsagittal T1-weighted MRI shows anatomically constrained medial territories; DLPFC, anterior insula, striatum, nucleus accumbens, amygdala, and hippocampal formation are identified as lateral or deep rather than falsely surface-mapped. The hypothalamus–pituitary relationship is separated from brainstem autonomic pathways and the VTA location is explicitly schematic. Functional convergence is probabilistic and does not define a sexual center. Anatomical background: OpenNeuro ds006072, version 1.1.0 (CC0) [36]. All colored overlays are author-controlled conceptual schematics, not quantitative lesion maps, statistical activation maps, or measurements of the anatomical-background participant. Their extent, position, and color do not encode lesion frequency, effect size, or a probability of sexual dysregulation.

Figure 1 summarizes how these interacting domains contribute to sexual regulation; their clinical relevance depends on the pattern of impairment and the context in which behavior occurs.

Prefrontal and cingulate systems contribute to value updating, conflict monitoring, consequence evaluation, and stopping; temporal-limbic systems contribute to salience, affective meaning, and contextual memory; the anterior insula contributes to interoceptive awareness; hypothalamic systems coordinate endocrine and autonomic components of arousal; and mesolimbic circuits support incentive salience and reinforcement learning [2,37,38,39,40,41,42,43,44,45,46,47,48]. These functions overlap and are domain-general. Their involvement does not imply that they encode a specific sexual preference.

Clinically, one possible mechanism is impaired coordination across systems. A patient may retain verbal knowledge of a rule yet fail to update value or inhibit behavior during fatigue, arousal, personal care, or ambiguous social interaction. Conversely, a patient may experience strong desire while maintaining contextual control. Neurobiological interpretation should therefore connect anatomy to an observable regulatory process rather than directly to a moral, diagnostic, or legal label.

### 2.2. Neurochemical, Cognitive, and Contextual Modifiers

Dopamine modulates incentive salience, reinforcement learning, and behavioral activation, whereas serotonin contributes to emotional and behavioral control; neither is a specific marker of sexual desire or preference [49,50,51]. Selective serotonin reuptake inhibitors (SSRIs) more commonly reduce orgasmic function and sexual satisfaction, although effects on desire vary [52]. Neurochemical explanations should therefore be framed as modifiers of distributed control rather than stand-alone causes.

Substance exposure can modify behavior independently of the lesion, but the evidence differs by agent. An acute cannabis fMRI pilot study found individually variable responses to erotic cues [53]. Intravenous methylphenidate increased reported sexual desire in a small non-ABI study [54], whereas alcohol experiments examined sexual risk-taking intentions [55]. These findings do not establish post-ABI effects. Sertraline-associated hypomania has been reported [31], so emerging mood activation warrants assessment. Noradrenergic arousal [56], oxytocin-related social salience [57], and hormonal modulation of desire [58] also require contextual interpretation. A clinically meaningful history must record agent, dose, timing, intoxication or withdrawal, and temporal relation to the behavior.

Executive regulation maintains a goal while an individual decides whether to act. Inhibition can interrupt an impulse, while flexibility allows behavior to change after feedback. Frontal and frontostriatal systems support these capacities [59,60,61]. Their disruption can leave verbal knowledge intact while its application during interaction fails.

Social cognition helps a person recognize another person’s refusal or discomfort and interpret interpersonal boundaries. Failure to use these signals can make sexual expression inappropriate without increasing desire [62,63]. Self-awareness is equally important: a systematic review after ABI associated impaired awareness with frontal and white-matter damage and altered connectivity involving anterior cingulate and inferior frontal systems, but did not examine sexual outcomes [64]. These findings support a regulatory mechanism, not a specific sexual-behavior localization.

Endocrine contributors require separate interpretation. A 2024 meta-analysis of 52 TBI studies (7367 participants) estimated pituitary-axis dysfunction in approximately one third of patients and gonadal deficiency in approximately one sixth [65]. Clinically, post-TBI hypopituitarism is therefore a plausible cause of fatigue, mood change, and reduced desire, but it should not be invoked as a default explanation for hypersexuality. Endocrine assessment should nevertheless consider conditions and treatments that may increase desire. Hyperthyroidism belongs in the differential, although its sexual effects are heterogeneous and increased libido should not be presumed: a prospective study in men documented sexual dysfunction, including reduced desire, in hyperthyroid patients [66]. Testosterone replacement may restore or increase libido in men with hypogonadism [67] and has been investigated during rehabilitation after TBI [68]. These findings do not establish that physiological replacement causes post-TBI sexual disinhibition. Clinical interpretation should relate any change in desire to thyroid status and the indication, dose, and timing of testosterone treatment, while distinguishing recovery of consensual sexual interest from impaired behavioral control. Neurochemical and endocrine pathways and their evidentiary limits are summarized in Supplementary Table S5.

The practical implication is to test competing explanations. Similar behavior may require different interventions depending on whether motivation or stopping is impaired. Mood activation and medication effects also require assessment. Table 1 summarizes the proposed functional substrates and limits of inference.

## 3. Sexual Dysregulation After Traumatic Brain Injury

### 3.1. Diffuse Network Injury and Executive-Control Vulnerability

Most post-TBI sexual problems involve reduced desire, dysfunction, fatigue, relationship strain, or unmet counseling needs [70,71,72,73,74,75,76,77,78,79,80,81,82]. Hypersexuality and disinhibition are less common but can substantially disrupt rehabilitation and caregiving. Intrusive touching may require an immediate response even without increased desire. Repetitive sexual activity may instead become problematic through persistence or its occurrence during shared care.

**Figure 2:**
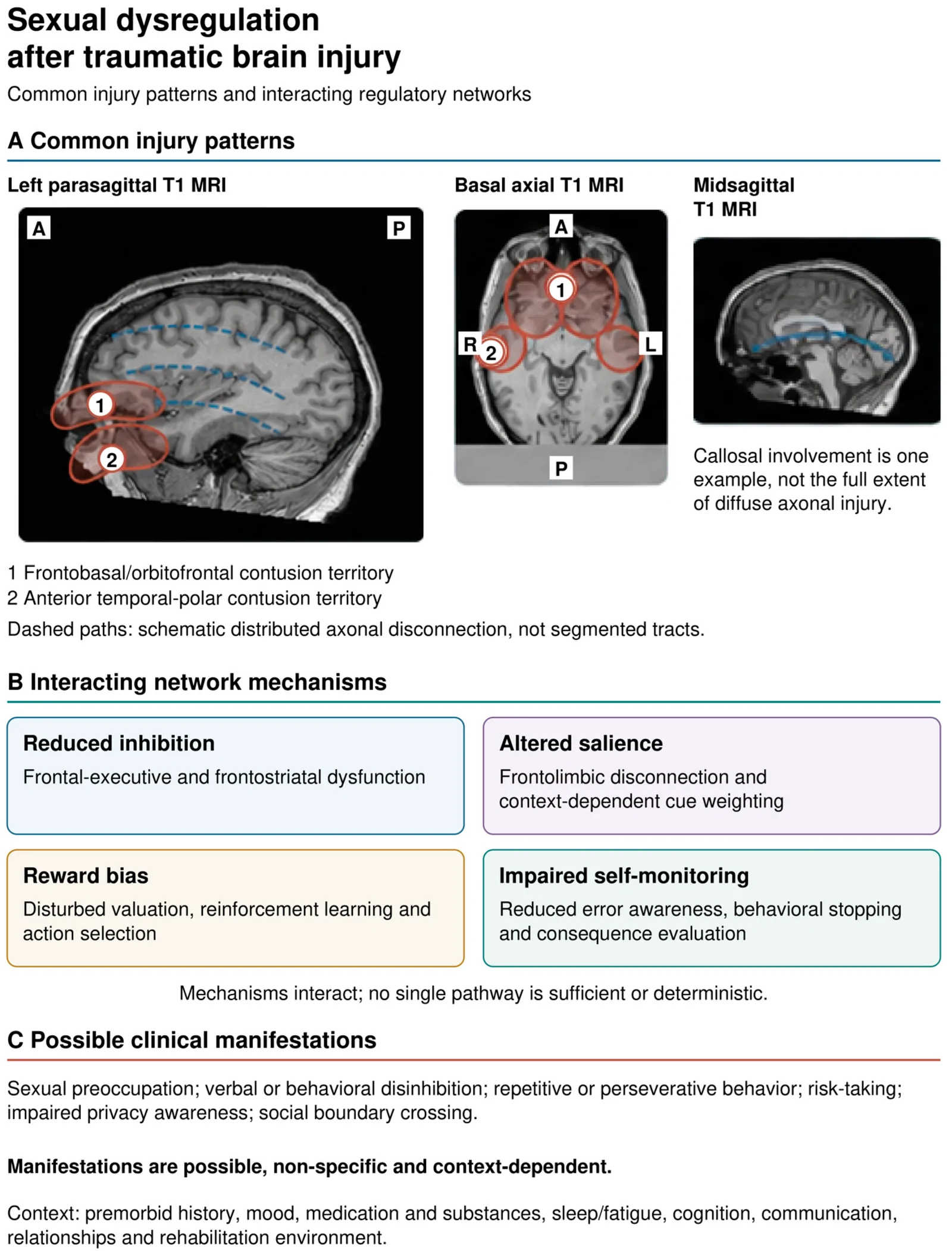
Network disruption model of sexual dysregulation after traumatic brain injury. Parasagittal and basal axial T1-weighted MRI views separate frontobasal/orbitofrontal from anterior temporal-polar contusion territories; dashed trajectories represent distributed axonal, frontostriatal, and frontolimbic disconnection schematically rather than focal lesion markers or tract segmentations. The interacting mechanisms and possible manifestations are non-specific, probabilistic, and modified by context. Common injury territories are not lesion-specific predictors of sexual behavior. MRI source: [36]. All colored overlays and dashed paths are author-controlled conceptual schematics, not quantitative lesion or connectivity maps derived from patients. Their extent and color do not represent measured lesion burden, tract damage, or a probability of the depicted behaviors.

The anatomical substrate of TBI is often distributed. Diffuse axonal injury can coexist with inferior frontal, orbitofrontal, ventromedial prefrontal, and anterior temporal contusions, disrupting frontostriatal and frontolimbic communication [83,84,85,86]. This pattern makes TBI more informative about network vulnerability than about a single causal site. Figure 2 distinguishes frontobasal/orbitofrontal from anterior temporal-polar contusion territories and depicts distributed axonal disconnection schematically rather than as focal lesion markers. Direct clinical evidence supports behavioral co-occurrence within a defined service population: the cross-sectional study by Simpson et al. included 507 people with severe TBI receiving care through 11 community-based rehabilitation services in New South Wales, Australia; 43 of the 45 participants with inappropriate sexual behavior also exhibited other challenging behaviors [18]. This association does not establish a uniform dysexecutive syndrome. Neither this co-occurrence nor the study’s prevalence estimate can be generalized to mild-to-moderate TBI, acute inpatient care, or people outside specialist rehabilitation services. Injury severity, service selection, observation opportunities, and informant reporting constrain transferability. Executive and social-behavioral studies provide a separate mechanistic basis for examining self-monitoring and response to feedback [87,88,89,90,91,92,93]. A large TRACK-TBI analysis also identified heterogeneous cognitive profiles at six months rather than a uniform impairment pattern [94]. Sexual behavior may become problematic because the patient cannot filter an impulse, recognize a cue, update contextual value, or anticipate interpersonal consequences; the proposed mechanism must therefore be tested against the individual’s cognitive profile, mood, medication, premorbid history, and setting.

Diffusion and functional-connectivity studies demonstrate widespread white-matter and network abnormalities after TBI [95,96,97]. Recent evidence reinforces both plausibility and heterogeneity: a systematic review of 50 resting-state fMRI studies found mixed significant and null results, with injury severity, chronicity, and imaging methods contributing to variation [98], while a small randomized imaging study found that methylphenidate altered corticostriatal connectivity alongside executive-function change [6]. Neither study measured sexual dysregulation. These findings therefore support candidate network mechanisms, not treatment efficacy or a lesion-behavior claim.

### 3.2. Clinical Phenotypes, Differential Formulation, and Rehabilitation Risk

Phenotyping should separate increased desire from impaired inhibition, frontal perseveration, poor privacy awareness, social-cue misreading, and compulsive repetition. Sexual comments to staff, for example, may reflect increased desire, but they may also arise from confabulation, poor filtering, or failure to appreciate the interpersonal setting. Repetitive masturbation can reflect boredom, habit, perseveration, or insufficient privacy rather than a primary paraphilic condition.

Case reports capture behavior that is difficult to study prospectively, but their interpretation depends on premorbid information and the course of recovery. In a five-case TBI series, several behaviors subsided without specific sexual-symptom medication, whereas one patient was lost to follow-up [26]. Spontaneous improvement and changes in supervision cannot be separated from the effects of redirection. Human Klüver-Bucy syndrome provides a further, mixed-etiology context: only two of the 12 patients in the landmark series had trauma, and altered sexual activity was recorded in seven [27]. It is therefore neither a general model for TBI nor a series of 12 post-traumatic sexual-dysregulation cases.

Acute confusional states, post-traumatic mania or hypomania, severe agitation, seizures, sleep disturbance, medication changes, substance exposure, and endocrine dysfunction can mimic or amplify sexual dysregulation [99,100]. Post-TBI hypopituitarism is common enough to warrant clinical attention when fatigue, reduced desire, menstrual change, erectile symptoms, or other endocrine features are present [65], but it more often explains reduced function than hypersexuality. Thyroid disease and testosterone exposure should also be reviewed when desire increases (Section 2.2). Persistent dysregulation should not be inferred until reversible contributors and temporal course have been evaluated.

Rehabilitation settings can expose regulatory vulnerability during personal care, when bodily exposure and dependence on staff complicate interpersonal boundaries. Shared rooms and unstructured time also change opportunity and observation. Patient, caregiver, and staff accounts should therefore be compared across these settings: disagreement may reflect different circumstances rather than an unreliable informant.

A TBI formulation should explain how the documented impairment produces an everyday difficulty. Redirection and comparison across settings can test that explanation, while the clinical timeline identifies modifiable contributors. The inference concerns impaired regulation rather than localization of a sexual behavior.

## 4. Sexual Dysregulation After Stroke

Stroke provides complementary evidence because a newly manifested phenotype can follow a focal or strategic vascular lesion. Broader sexual dysfunction is common: contemporary reviews and guidance emphasize reduced desire, arousal, orgasm, satisfaction, and relationship effects rather than dysregulation [13,101,102,103,104]. Frontal, temporal-limbic, basal-ganglia, insular, thalamic, or hypothalamic involvement may be anatomically informative in rare cases, but hypersexuality and sexual disinhibition remain uncommon [105,106,107,108]. Temporality strengthens hypothesis generation; it does not establish deterministic causation.

### 4.1. Strategic Lesions and Regulatory Network Nodes

Frontal lesions are plausible contributors because orbitofrontal, ventromedial prefrontal, medial frontal, and cingulate systems support inhibition, value updating, and consequence evaluation [2,39,40,41,42]. Disinhibited comments, advances, or exposure can therefore occur without increased libido. The evidence does not support a simple right-hemisphere rule; laterality is modified by lesion size, disconnection, aphasia, neglect, premorbid traits, and reporting.

Temporal-limbic injury may alter affective salience, distress-cue recognition, or the contextual and relational meaning of behavior [28]. Because most reports lack standardized measurement of premorbid sexual interests and longitudinal follow-up, these lesions should not be interpreted as direct markers of altered preference.

**Figure 3:**
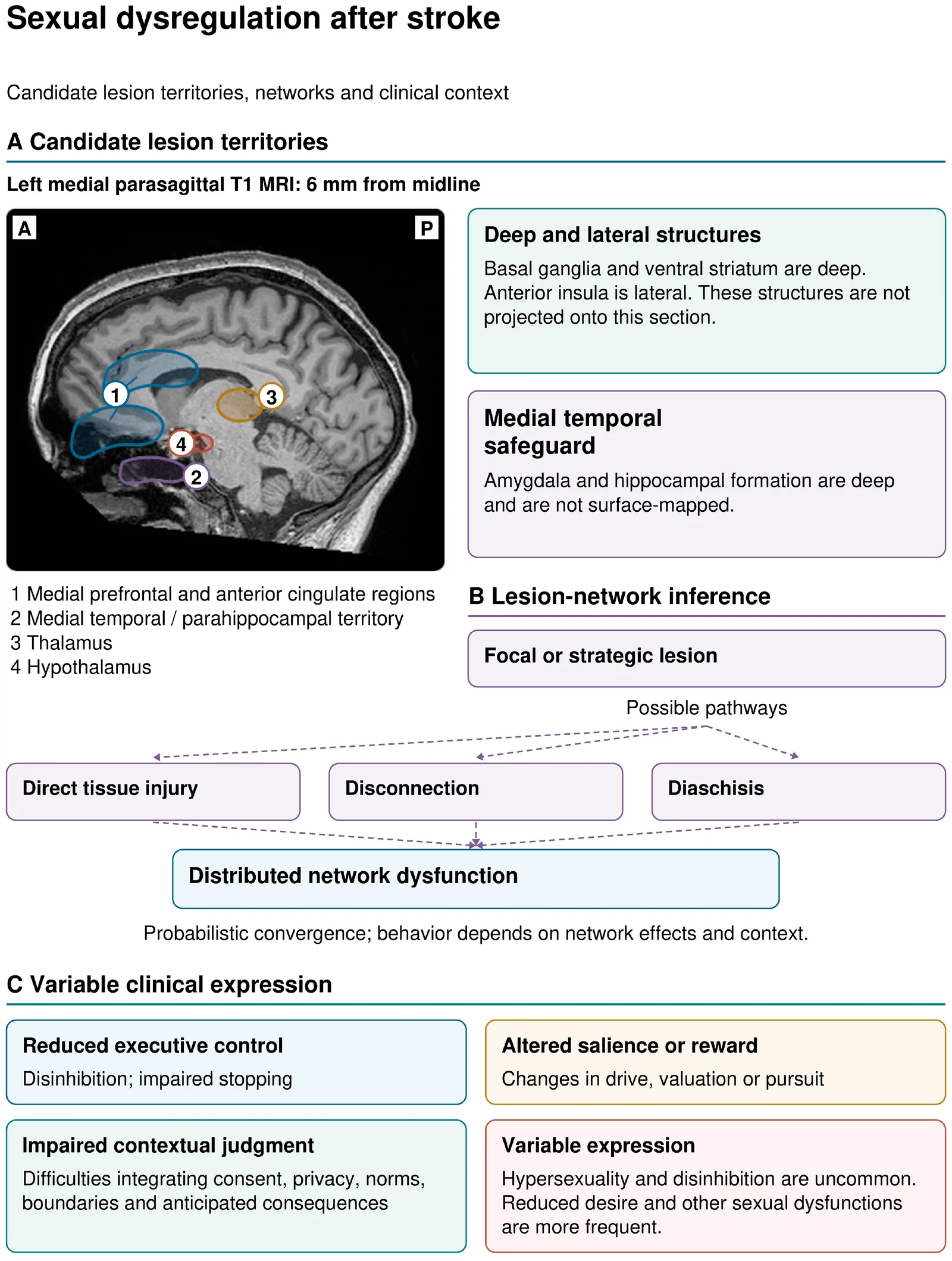
Lesion-network model of sexual dysregulation after stroke. A left medial parasagittal T1-weighted MRI (6 mm from the midline) distinguishes medial prefrontal/anterior cingulate, medial temporal/parahippocampal, thalamic, and hypothalamic candidate territories. Amygdala, hippocampal formation, basal ganglia, ventral striatum, and anterior insula are listed as deep or lateral and are not surface-mapped. Direct injury, disconnection, and diaschisis converge probabilistically; lesion location does not determine behavior. Temporal association does not establish a newly acquired preference. Premorbid history, mood, medication, aphasia, anosognosia, observer context and environment modify expression; imaging alone cannot determine intent, responsibility, consent capacity or legal conclusions. MRI source: [36]. All colored overlays are author-controlled conceptual schematics, not quantitative lesion maps, statistical activation maps, or patient-specific lesion segmentations. No voxelwise lesion-symptom or lesion-network analysis was performed for this figure; color and extent do not quantify behavioral risk.

The thalamus links frontal, limbic, attentional, and arousal systems. Spinella described hypersexuality with a clinically inferred paramedian thalamic syndrome after intraventricular hemorrhage; follow-up MRI did not demonstrate an infarct [17]. In contrast, Mutarelli et al. documented bilateral paramedian infarction on MRI and frontal hypoperfusion on SPECT, with persistent hypersexuality despite normal structured executive testing [29]. Broader thalamic and basal-ganglia studies inform candidate mechanisms but do not establish sexual-outcome frequency or specificity [109,110,111]. Hypothalamic injury may affect physiological arousal, although direct evidence for complex behavior is sparse. Figure 3 distinguishes candidate medial territories from deep or lateral structures.

Recent lesion-network studies outside sexual outcomes also argue for restraint. In 565 patients with ischemic stroke, lesion-network mapping reproduced symptom topography but did not improve prognostic prediction beyond clinical variables [112]. Across two prospective cohorts, lesion location was not associated with post-stroke depressive symptoms and network associations were weak or non-replicated [113]. These studies do not identify sexual networks; they show why connectome-based localization must be validated prospectively and should not substitute for phenotype-specific data.

### 4.2. Heterogeneous Phenotypes, Confounders, and Anatomical Caution

Post-stroke case reports describe increased sexual activity or initiation and impaired behavioral control [17,29]. These observations concern heterogeneous presentations and cannot establish a typical post-stroke phenotype. Observable, phenotype-specific terms are preferable to ‘deviant sexual behavior’, which conflates mechanism, morality, diagnosis, and risk.

Case reports offer temporality and anatomical detail but are selected for novelty. Premorbid characterization and follow-up determine how much can be inferred. In the Mutarelli case report, hypersexuality persisted for 9 years despite improved memory [29]. Preserved structured executive-test performance in this patient challenges an account that assigns primary responsibility to executive deficits. Such tests may not capture context-dependent social or motivational difficulties, but unmeasured impairment cannot be assumed. Frontal hypoperfusion is compatible with network involvement; the original authors also acknowledged that it might be unrelated to hypersexuality [29]. The case therefore leaves altered drive, salience, and behavioral control as competing or interacting hypotheses rather than establishing a predominantly dysexecutive mechanism. These observations warrant clinically specific accounts rather than a uniform post-stroke syndrome.

Post-stroke mania or hypomania is a key differential because it can increase libido, talkativeness, impulsivity, reduced sleep, irritability, and risk-taking [114,115]. Delirium, severe aphasia, neglect, and anosognosia can also change behavior or make intention difficult to assess. These conditions are assessment modifiers, not alternative moral explanations.

Medication-related activation also requires explicit exclusion. The medication history should cover dopamine agonists, such as pramipexole or ropinirole, and other dopaminergic or catecholaminergic agents, including levodopa-containing preparations and stimulants, whether prescribed for a comorbidity or a rehabilitation indication. Dopamine agonist exposure is associated with impulse-control disorders, including compulsive sexual behavior, in Parkinson disease; extrapolation to stroke identifies a safety differential, not a post-stroke incidence estimate [116]. Levodopa has been studied as a rehabilitation adjunct, but the DARS trial did not demonstrate improved independent walking and does not establish a sexual-behavior effect [117]. New behavior after initiation or dose escalation should prompt review of the indication and temporal relationship with the prescriber; clinically appropriate supervised adjustment and reassessment may clarify contribution. Deliberate re-exposure solely to prove causality is not warranted.

Timing and persistence are informative. Behavior confined to acute confusion requires reassessment after stabilization. Later onset warrants review of mood, treatment, and changes in daily life. Future reports should connect lesion findings to a premorbid baseline and repeated behavioral assessment, while evaluating capacity and consent separately.

## 5. Rare Acquired Paraphilic Manifestations

Acquired paraphilic manifestations are rare and require heightened care because assessment intersects with intimate privacy, consent, safeguarding, stigma, possible harm to others, and legal proceedings. Diagnostic frameworks distinguish paraphilic interest, enacted behavior, and paraphilic disorder; after ABI, the same observation may instead reflect disinhibition, impaired privacy awareness, compulsive repetition, medication effects, or opportunity [118,119,120,121]. Supplementary Table S6 summarizes reported contexts while preserving these diagnostic and causal safeguards.

### 5.1. Diagnostic Boundaries and Phenotypic Ambiguity

Reports describe varied atypical sexual content, but these presentations are exceptional. ‘Acquired paraphilic manifestation’ is most useful provisionally when a credible temporal relationship with neurological change is established. Its meaning depends on the observation: a newly documented act may be well supported while a change in enduring interest remains uncertain [118,119,120,121]. Premorbid information and persistence therefore determine the strength of the inference. A lesion or neurobehavioral syndrome alone cannot establish a new sexual preference.

An inappropriate act does not necessarily demonstrate an enduring preference: impaired inhibition may expose a pre-existing interest, while stimulus-bound behavior may occur without a stable change in interest. Available reports rarely provide sufficient premorbid or longitudinal information to resolve this distinction [118,119,120,121]. A broader dysexecutive or neuropsychiatric syndrome must be considered before attributing the observed content to a new preference.

Clinical assessment should establish a temporal account of change, using premorbid sexual and relationship history when ethically obtainable. Reported fantasies or urges should be distinguished from observed conduct, and the response to redirection should be documented. The reporting source matters because patients and partners may describe experiences that are not visible to clinical staff. Staff may instead observe difficulties specific to personal care. Decision-specific consent capacity requires separate assessment, with communication support where necessary; cognitive impairment may limit the reliability of self-report without making every account uninformative.

Potential alternative or contributing explanations require systematic evaluation, particularly when behavior begins during confusion, mood activation, or a medication change. The differential assessment described in Section 7.1 helps establish whether the apparent phenotype persists after these contributors are addressed. Institutional routines can also make behavior observable without causing it. Repeated assessment across settings and after medical stabilization is therefore preferable to assigning a definitive label on the basis of a single episode.

Particular caution is warranted when behavior involves vulnerable persons, coercion, or potentially unlawful conduct. Clinical documentation should describe observable behavior, context, capacity, risk, and uncertainty without inferring criminal intent, legal responsibility, or a stable paraphilic identity from neurological findings alone. A proportionate formulation should support safeguarding and risk management while avoiding unnecessary stigmatization. Where uncertainty persists, the most defensible description is phenotype-based and mechanism-informed, for example, “newly observed atypical sexual behavior associated with impaired inhibition and contextual judgment”, rather than a categorical causal diagnosis.

### 5.2. Fronto-Temporal Mechanisms, Forensic Caution, and Safeguarding

Fronto-temporal dysfunction is a plausible shared vulnerability because prefrontal control and temporal–limbic salience systems interact [20,30,122]. The tumor reports provide particularly informative within-person sequences: symptoms improved after resection, and Burns and Swerdlow also observed renewed behavioral difficulty with tumor regrowth [20,30]. These observations support temporal association but involve mass effects across connected structures. Their role is to inform mechanisms; they do not establish TBI/stroke treatment efficacy or prove that a lesion created a specific sexual preference.

Pedophilic manifestations require the greatest caution. Some cases may represent generalized disinhibition, hypersexuality, impaired moral-emotional judgment, or reduced appreciation of harm rather than a selective and stable alteration in sexual interest. Safeguarding obligations remain independent of the proposed neurological mechanism.

Exhibitionistic, voyeuristic, and frotteuristic behavior may be more parsimoniously formulated through disinhibition, privacy unawareness, reduced empathy, or poor recognition of another person’s discomfort. Population and neurological literature reinforces the need to separate atypical interest, behavior, disorder, and offending [123,124,125].

In evaluations conducted for legal proceedings, neuroscience can inform risk formulation and treatment planning but cannot determine intent, responsibility, or a legal conclusion. Capacity, consent, appreciation of harm, impulse control, and legal accountability are distinct questions. Documentation should show how the observed cognitive difficulties relate to the particular behavior and what remains uncertain. A lesion alone cannot explain the act or settle the legal questions.

Most people with TBI or stroke do not develop paraphilic behavior, and most sexual offending is not explained by a newly acquired neurological syndrome. Comparisons with frontotemporal syndromes and acquired sociopathy can clarify loss of empathy, social control, or reward regulation, but remain analogical rather than direct evidence [126,127,128].

## 6. Cross-Etiology Synthesis: What TBI and Stroke Can and Cannot Show

TBI informs distributed regulatory vulnerability, whereas stroke can nominate lesion-connected nodes. Their comparison is useful when it clarifies the relationship between an observed sexual phenotype and the regulatory functions that may be impaired. An intrusive act associated with poor self-monitoring cannot automatically be equated with increased desire, even when both reports use the same label. Likewise, the appearance of sexually inappropriate behavior does not, by itself, establish a change in enduring sexual preference. Distinguishing motivation from its behavioral expression is essential before apparently similar observations are assigned a shared mechanism [14,18,19,29].

Cross-etiology synthesis also requires attention to the circumstances in which behavior becomes observable. A pattern documented during supervised rehabilitation may not be directly comparable with behavior reported after community discharge, when opportunities for expression and sources of information differ. The timing of observation also matters: behavior first reported after an injury may have begun earlier or become more apparent under changed circumstances. The observation timeline should therefore be distinguished from the inferred onset of the behavior.

**Figure 4:**
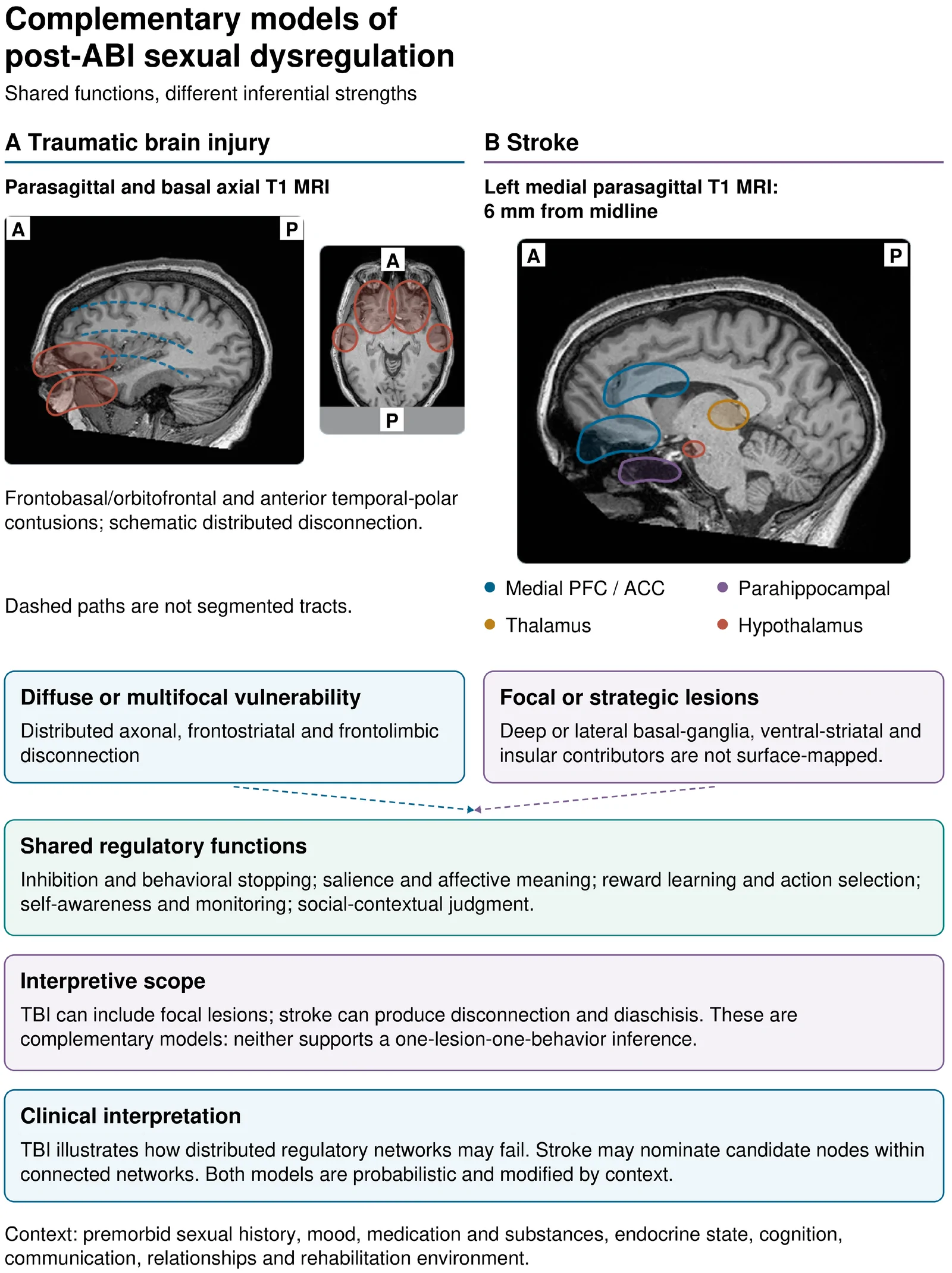
Complementary models of post-ABI sexual dysregulation. Parasagittal and basal axial T1-weighted MRI views depict common TBI contusion and distributed disconnection patterns, whereas a left medial parasagittal T1-weighted MRI (6 mm from the midline) depicts restrained stroke candidate territories. Both etiologies converge on shared regulatory functions rather than a single anatomical hub. TBI can include focal lesions and stroke can produce disconnection and diaschisis; neither supports a one-lesion-one-behavior inference. Contextual modifiers include premorbid sexual history, mood, medication and substance exposure, endocrine state, cognition, communication, relationships and the rehabilitation environment. MRI source: [36]. Colored overlays are author-controlled conceptual schematics rather than quantitative lesion maps; no patient-level lesion, tract, or behavioral-risk measurements are encoded.

At the mechanistic level, similar manifestations across etiologies may suggest disruption of a shared regulatory function, but they do not demonstrate an identical anatomical substrate. Conversely, variation in behavior among patients with apparently similar injuries highlights the limits of anatomical interpretation without adequate clinical characterization. The value of comparison lies in identifying convergent observations while preserving these distinctions. Figure 4 integrates the complementary perspectives offered by TBI and stroke, together with the contextual factors and inferential limits governing their interpretation.

### 6.1. Complementary Inferential Strengths

In TBI, diffuse axonal injury, contusions, and disconnection can weaken regulation across fatigue, personal care, reduced privacy, or interpersonal ambiguity. A patient may verbalize a rule in a structured interview yet fail to apply it during an ongoing interaction; this is impaired real-time behavioral regulation, not proof of increased desire [129,130,131].

In stroke, a close temporal relationship between a strategic lesion and new behavior can nominate a regulatory node. Connected-network effects may explain more than the visible lesion alone; premorbid information and clinical context determine whether that hypothesis fits the phenotype [69,132,133,134].

The models are therefore complementary: focal anatomy generates candidate nodes, network approaches test connected systems, and clinical formulation determines whether the proposed mechanism matches the phenotype. TBI can include focal lesions, and stroke can produce white-matter disconnection and remote network dysfunction; a strict focal-versus-diffuse dichotomy is misleading.

Diffuse-network accounts can become too broad to falsify when sexual outcomes were not measured [6,85,86,87,88,89,90,91,92,93,95,96,97,98]. Focal accounts may overattribute behavior to the visible lesion [109,110,111,114,115]. Lesion-network methods connect these approaches, but findings require validation against a defined phenotype in independent samples before clinical use [69,112,113,132,133,134]. Table 2 summarizes these trade-offs.

### 6.2. Evidence Quality, Terminology, and Reporting Bias

TBI studies often provide rich rehabilitation and caregiver context but weak lesion specificity; stroke reports may offer striking anatomy but are dominated by rare, publication-prone cases. Neither evidence stream supports incidence estimates or deterministic localization. The strongest conclusion is usually conditional: a documented injury may increase vulnerability to a defined phenotype under particular cognitive, psychiatric, relational, and environmental conditions.

For rare phenotypes, evidentiary value depends on the quality of within-person change. A corroborated premorbid baseline makes the timing of onset interpretable, especially when injury and medication changes occur close together. Follow-up should test persistence across settings and after treating competing explanations. Confidence falls when evidence is limited to a single observer’s account or when potentially reversible contributors remain unassessed. Selective publication of striking or legally salient presentations can exaggerate the apparent coherence of an anatomical explanation. A missing premorbid history was treated as unknown, not as evidence that an interest was absent before injury. Accordingly, a newly observed act, a newly reported interest, and a demonstrably new enduring preference were not treated as equivalent findings. This interpretive handling limits overattribution but cannot remove publication bias or recover unpublished cases.

Terminology remains a major source of bias. ‘Hypersexuality’ is sometimes used for increased desire and sometimes for behavior that is difficult to inhibit. This ambiguity obscures whether the clinical problem concerns drive, control, or the setting of expression. Table 3 therefore separates phenotypes by observable features, competing mechanisms, and differential considerations.

Causal inference should be graded by the claim being made. TBI cases more often support a claim about regulatory vulnerability; stroke cases may support a claim about a candidate node when onset is clear and confounders are addressed. Neither supports inference about preference, intent, or responsibility without separate clinical evidence.

Real-world validity concerns whether structured assessment predicts behavior during everyday care and social interaction. Staff and caregiver observations are indispensable but are not neutral: burden, fear, relationship history, ward culture, the observer’s gender and role, and differing thresholds for documenting male, female, or gender-diverse patients can shape reporting. These influences should be recorded rather than treated as noise.

Future evidence should therefore connect anatomy to repeated observation of a clearly defined phenotype. Recording who observed the behavior and the opportunity for its expression is necessary to interpret change over time, alongside neuropsychological assessment and review of treatable contributors.

## 7. Clinical Assessment, Risk Formulation, and Management Pathways

Clinical care begins by describing the behavior and evaluating acute or treatable contributors. The resulting formulation should guide a targeted intervention, within the limits set by decision-specific capacity and safeguarding. Monitoring then determines whether to continue, adjust, or de-escalate treatment. This sequence is a clinical synthesis, not a formal treatment guideline.

### 7.1. Descriptive Assessment and Mechanism-Based Formulation

For multidisciplinary use, the assessment can be organized into four linked steps: behavioral phenotyping, medical and pharmacological exclusion, capacity evaluation, and environmental risk assessment. This is a proposed clinical assessment pathway, not a validated diagnostic algorithm. Immediate protection from harm proceeds in parallel, and the sequence is revisited when cognition, treatment, or the care setting changes.

#### 7.1.1. Behavioral Phenotyping

Assessment begins with a description of what happens, when it began, and how it differs from baseline. Recording the circumstances and response to redirection helps define a measurable target [136,137]. Increased libido, disinhibition, compulsive repetition, intrusive thoughts, context-inappropriate behavior, nonconsensual contact, and atypical interest should be documented separately [139,140]. A sexualized comment during bathing, for example, requires assessment of exposure, dependence, communication, and privacy before it is labelled as hypersexuality.

Supplementary Table S7, Panel B, provides a proposed minimum observation log. Teams should agree on neutral operational definitions and the observation interval, record both events and adequately observed intervals without events, and distinguish absence of behavior from absence of observation. Patient-reported desire, distress, and sexual-function difficulties should remain separate fields from staff-observed acts. Antecedents, medication timing, redirection, and consequences permit within-person comparison, while observer role and opportunity help interpret differences across settings. The log is intended to support consistent documentation within a protocol; it is not a validated scale, diagnostic threshold, or substitute for clinical assessment.

Measures of sexual function such as the ASEX, CSFQ, IIEF, and FSFI can characterize selected domains of sexual function [141,142,143,144], but were not designed to diagnose post-ABI sexual disinhibition. Distress and relationship impact require complementary clinical assessment. Their use should match communication ability, sex-related anatomy, clinical aim, and patient preference rather than function as a generic battery.

The Hypersexual Behavior Inventory, Sexual Compulsivity Scale, and CSBD-DI may inform compulsive or hypersexual symptoms, while the NPI, FrSBe, Barratt Impulsiveness Scale, and TASIT can characterize broader behavioral and social–cognitive deficits [145,146,147,148,149,150,151]. None is validated as a stand-alone measure of post-ABI sexual dysregulation, and self-report may be distorted by aphasia, memory impairment, anosognosia, or restricted opportunity.

Neurorehabilitative formulation should establish whether motivation, regulation, or opportunity is the principal difficulty [152,153,154,155,156]. Capacity and risk cannot be inferred from impulsivity alone. Formal testing may miss problems arising during fatigue or dependence on staff; assessment therefore needs observations outside the interview.

The patient’s account should be obtained with appropriate communication support and compared with caregiver and staff observations. Each report should identify the observer and setting. Differences may reveal situational triggers; the reporting biases discussed in Section 6.2 should inform interpretation.

Neuropsychological assessment should focus on response inhibition, flexibility, awareness, social cognition, emotion recognition, and decision-making, with structured behavioral observation across settings [152,153,154,155,156]. Intrusive urges with preserved insight require a different pathway from boundary-crossing behavior accompanied by severe anosognosia.

#### 7.1.2. Medical and Pharmacological Exclusion

The differential diagnosis should follow the timing and accompanying symptoms. Acute confusion warrants evaluation for delirium, seizures, or substance effects; later change may be associated with mood disturbance, medication exposure, or endocrine dysfunction [65,99,100,114,115,140]. Communication impairment and poor sleep can further complicate interpretation. For example, low libido with fatigue after TBI warrants pituitary review, whereas new risk-taking with reduced sleep and pressured speech requires assessment and treatment of mania before lesion-based attribution. Endocrine assessment should include thyroid function when clinically indicated, and medication reconciliation should explicitly document testosterone replacement and other androgen exposure [66,67,68]. Behavior should be reassessed after stabilization or a relevant treatment change before a persistent sexual phenotype is assigned.

Medication reconciliation should include prescribed, as-needed, recently discontinued, and non-prescribed agents. Record the indication, dose change, onset of behavior, sleep and mood change, and response after any supervised adjustment; the specific dopaminergic differential is discussed in Section 4.2. A plausible exposure-behavior sequence supports further assessment but does not establish causality, particularly when delirium, recovery, or changes in supervision occur concurrently.

#### 7.1.3. Capacity Evaluation

Assess capacity for the particular decision at the relevant time, with accessible communication and support for comprehension. Capacity to consent to treatment or research must not be used as a proxy for capacity concerning sexual activity, and a cognitive score or aphasia alone cannot settle either question. Document understanding and appreciation of the relevant situation, ability to communicate a voluntary choice, recognition of another person’s agreement or refusal, and possible coercion or exploitation. The applicable legal framework and clinical context determine the precise standard. Reassess after delirium or mood stabilization and when circumstances change; unresolved concern warrants specialist input and proportionate protection [157,158].

#### 7.1.4. Environmental Risk Assessment

Map where and with whom behavior occurs, including personal care, shared rooms, unstructured time, fatigue, communication mismatch, and access to privacy. Identify specific risks to the patient or others, while distinguishing consensual expression from intrusive or nonconsensual conduct. Agree on consistent redirection, appropriate private space, meaningful activity, caregiver/staff coaching, and the least restrictive supervision that addresses the documented risk. Record a named professional responsible for review and measurable criteria for increasing or reducing support. The resulting formulation should link each intervention to an observed trigger or difficulty and protect opportunities for appropriate intimacy [159,160,161,162].

A phenotype-first formulation also changes immediate management. Repetitive masturbation on a ward may reflect boredom, perseveration, or absent privacy and may respond first to structured activity and scheduled private time. Improvement supports continued environmental management and reassessment. These examples identify testable mechanisms and proportionate interventions without minimizing safeguarding.

### 7.2. Safeguarding, Treatment Options, and Preservation of Intimacy

Environmental and behavioral measures are usually the first intervention because many incidents are setting-dependent. Counseling should be adapted to comprehension, with visual cues when verbal explanations are insufficient. Scheduled privacy and structured routines may reduce situational triggers; consistent staff responses and caregiver coaching help maintain the same approach across settings [159]. Recent TBI guidance recommends early, individualized sexuality education and multidisciplinary support, although most recommendations are consensus-based [21]. Immediate risk requires safeguarding before broader rehabilitation goals.

Sexual decision-making capacity is decision- and context-specific. Assessment must establish understanding, appreciation, and voluntary choice, while considering vulnerability and power imbalance [157,158]. Safeguarding is required when others face harm; unresolved conflicts between risk, rights, and capacity may require ethics or legal consultation.

No direct randomized treatment evidence for post-ABI sexual dysregulation was identified in the sources synthesized here. A recent mixed-method co-design and implementation study in a TBI rehabilitation unit reported improved sexuality support, help-seeking, and clinician attitudes after service changes, but it evaluated implementation rather than efficacy for hypersexuality or disinhibition [22]. Table 4 therefore grades each intervention separately by directness and the 2011 OCEBM treatment-benefit hierarchy [34]; implementation outcomes are not presented as proof of a sexual-dysregulation treatment effect.

SSRIs may be considered when intrusive sexual thoughts, compulsive features, depression, anxiety, or impulsivity are plausible targets, but supporting studies largely involve non-ABI compulsive sexual behavior or paraphilic disorders [163,164,165,166,167]. Post-ABI use is therefore indirect or case-based and requires monitoring for sexual dysfunction, agitation, and rare activation or hypomania [31].

Mood stabilizers and antipsychotics should target mania, psychosis, severe agitation, or impulsive-aggressive dyscontrol rather than sexual behavior per se [115,168,169,170]. Evidence for a sexual outcome is indirect, and successful treatment of an underlying syndrome should not be interpreted as proof of a lesion-specific sexual mechanism.

Antiandrogenic or hormonal treatment should be exceptional and limited to persistent high-risk behavior after interdisciplinary review. Britton reported improvement with weekly intramuscular medroxyprogesterone after severe TBI, but the uncontrolled sequence and inconsistent delivery of the preceding behavioral program limit attribution [15]. Broader recommendations derive from non-ABI paraphilic-disorder literature [166,167,171,172,173,174,175]. Any such treatment requires consent/capacity review and specialist monitoring of endocrine, bone, metabolic, and other adverse effects. Naltrexone evidence remains indirect: the cited non-ABI cases also received serotonin reuptake inhibitors, preventing isolation of the drug’s effect [32,33].

Every intervention should be a monitored, reversible trial with a prespecified target. Baseline observations should define benefit in terms of incident frequency, redirectability, and participation. A quieter ward does not establish success if it reflects excessive sedation or lost intimacy. Monitoring should therefore address adverse effects and the experience of patients and caregivers as well as safety [160,161,162]. Lack of benefit or changing risk should trigger de-escalation. Medication used primarily to suppress behavior through sedation, or blanket restrictions on contact, privacy, or intimacy, should not be the default response. Any temporary restriction requires a documented immediate risk, the least restrictive feasible alternative, a responsible clinician, a review time, and criteria for relaxation or cessation. Monitoring must consider falls, confusion, cognitive slowing, distress, functional participation, and loss of appropriate intimacy alongside incident counts. Apparent improvement attributable to reduced opportunity or excessive sedation is not sufficient therapeutic benefit; an unfavorable benefit–harm balance requires prompt reassessment and supervised adjustment.

Safety planning must not become blanket suppression of sexuality. When capacity permits, the patient’s goals, values, privacy, partnership, contraception needs, and sexually transmitted infection education should be addressed alongside risk. Partners may require support for consent, changed roles, emotional adjustment, and communication. Ethical neurorehabilitation protects others while preserving lawful, consensual intimacy.

**Table 4:** Evidence-graded assessment and management framework for post-ABI sexual dysregulation.

Clinical Domain or Intervention	Clinical Use or Target	Best Direct Post-ABI Evidence (OCEBM)	Indirect Evidence and Directness	Safeguards and Interpretation
Behavioral description	Define phenotype, onset, setting, triggers, redirectability, affected persons, and consequences.	Not a treatment question.	Not applicable.	Use observable language; avoid stigmatizing labels and premature diagnosis. Sources: [71,139,159,160]
Neuropsychological and contextual assessment	Assess inhibition, awareness, social cognition, communication, decision-making, and behavior across settings.	Not a treatment question.	Not applicable.	Structured tests may miss boundary problems during personal care or unstructured interaction. Sources: [140,153,154,155]
Treatment of an active contributing syndrome	Treat mania, psychosis, delirium, seizures, severe agitation, pain, sleep disturbance, or substance-related states, then reassess.	Level 4–5 for sexual outcomes: case-based or mechanism-based.	Evidence primarily concerns the underlying syndrome rather than post-ABI sexual dysregulation.	Do not infer lesion-specific sexual efficacy from improvement after syndrome treatment. Sources: [99,114,115,140,168,169,170]
Medication and endocrine review	Identify dopaminergic, stimulant, sedative, antidepressant, antiepileptic, hormonal, or pituitary contributors.	Not a treatment-effect question.	Mechanistic evidence is indirect; post-TBI endocrine prevalence estimates support assessment of clinically compatible symptoms [65].	Record medication dose and timing [31,53,54,55,168,169,170]. Assess fatigue, libido, mood, arousal, menstrual or erectile symptoms, and pituitary function when indicated [58,65].
Psychoeducation and caregiver/staff training	Improve shared terminology, consistent responses, privacy planning, communication, and early recognition of risk.	Level 3 for implementation/service outcomes; Level 5 for direct sexual-dysregulation efficacy.	A TBI guideline is predominantly consensus-based; one mixed-method implementation study supports service delivery rather than dysregulation-treatment efficacy [21,22].	Provide early individualized education, written materials, relationship support, and trained clinical champions; include patient goals and avoid treating collateral reports as neutral. Sources: [21,22,70,105,106,107,108,135,152,156]
Consent, capacity, and safeguarding	Assess decision-specific understanding, appreciation, reasoning, voluntariness, communication, vulnerability, and risk.	Not a treatment-effect question.	Capacity and ethics literature informs practice indirectly.	Protect vulnerable persons while preserving dignity and lawful intimacy. Sources: [157,158]
Behavioral and environmental management	Modify antecedents, privacy, routines, cues, reinforcement, supervision, and staff responses.	Level 4–5: selected case-based observations plus mechanism-based reasoning.	Broader ABI behavioral intervention evidence is indirect for sexual outcomes.	Adapt to cognitive profile; define a target and monitor burden and restriction. Sources: [159,160,161]
Selective serotonin reuptake inhibitors	Target intrusive sexual thoughts, compulsive features, depression, anxiety, or selected impulsive symptoms.	No direct comparative post-ABI evidence identified in the cited sources.	The cited sexual-symptom treatment studies concern non-ABI populations; post-ABI use is an extrapolation.	Monitor sexual dysfunction, agitation, and rare activation or hypomania. Sources: [31,163,164,165,166,167]
Interdisciplinary rehabilitation and intimacy support	Integrate neurology, psychiatry, psychology, nursing, rehabilitation, social work, sexual health, ethics, and caregivers.	Level 3 for implementation/service outcomes; Level 5 for direct sexual-dysregulation efficacy.	Guideline and co-design evidence supports organized sexuality care, but direct efficacy for hypersexuality or disinhibition remains untested [21,22].	Use trained champions and referral pathways; risk management should not become blanket suppression of sexuality or partnership goals. Sources: [11,12,21,22,70,105,107,152,156]
Psychotherapy and collaborative coping	Address distressing urges, shame, adjustment, communication, coping, relapse planning, and preserved intimacy when insight permits.	Level 5: no direct controlled post-ABI sexual-dysregulation evidence identified.	Psychological evidence is extrapolated from adjacent conditions and ABI adjustment literature.	Adapt to awareness, memory, communication, and capacity. Sources: [152,153,154,155,156]
Mood stabilizers or antipsychotics	Target mania, psychosis, severe agitation, or impulsive-aggressive dyscontrol, not sexual behavior in isolation.	Level 4–5 for sexual outcomes: syndrome-specific cases or mechanism-based reasoning.	ABI agitation and psychiatric evidence is indirect for sexual outcomes.	Monitor cognition, sedation, metabolic effects, and participation. Sources: [115,168,169,170]
Antiandrogenic or hormonal treatment	Consider only exceptional, persistent, high-risk behavior after interdisciplinary review.	Level 4: direct ABI case-report evidence; no controlled ABI evidence.	Most guidance derives from non-ABI paraphilic-disorder literature.	Require capacity/consent review, endocrine input, bone and metabolic monitoring, and ethical safeguards. Sources: [15,166,167,171,172,173,174,175]
Naltrexone	Possible specialist option for selected reward-driven or compulsive patterns.	No direct post-ABI evidence identified.	Level 4 indirect evidence from non-ABI case reports.	Use only with an explicit target, contraindication review, and outcome monitoring. Sources: [32,33]

Abbreviations: ABI, acquired brain injury; OCEBM, Oxford Centre for Evidence-Based Medicine. OCEBM levels refer to the best evidence directly addressing treatment effects on post-ABI sexual dysregulation; they do not constitute recommendations.

## 8. Discussion, Novelty, and Research Priorities

This review treats post-ABI sexual dysregulation as a heterogeneous disturbance of sexual motivation, behavioral control, or their interaction. Anatomical explanations are useful when they change the clinical question being tested. Transient recovery-phase behavior and persistent post-stroke symptoms should not be combined as one outcome. Direct treatment evidence remains weak; broader ABI studies commonly assess agitation, cognition, or sexuality support rather than dysregulated sexual outcomes [21,22,32,33,160,161,162,163,164,165,166,167,168,169,170,171,172,173,174,175,176].

### 8.1. Novelty and Practical Contribution

The practical contribution is to connect phenotypic distinctions to the strength of the evidence supporting a clinical decision. The TBI–stroke comparison and anatomical figures make the limits of localization explicit. This approach links mechanism to assessment and management without treating plausibility as proof of treatment benefit. Research priorities are summarized in Supplementary Table S7, while Supplementary Table S8 maps the directness and limitations of the recent evidence update.

Compared with contemporary sexual-health reviews and guidelines, this synthesis focuses on less common dysregulation without presenting it as the typical sexual consequence of ABI [13,21,74,75,76,77,78,79,80,81,82,100,101,102,103,104]. Compared with case-based anatomical accounts, it defines the inferential boundary: TBI can illustrate distributed regulatory vulnerability, whereas stroke can nominate candidate nodes whose expression still depends on network and clinical context. Connectivity and lesion-network studies support this systems logic but remain mechanistic, not direct validation, because they did not measure sexual dysregulation [6,98,112,113].

Clinical use begins with a sequence of discriminating questions. Is desire increased, or is stopping impaired? Does behavior occur across settings or only during personal care, boredom, fatigue, or reduced supervision? Is the patient aware and redirectable? Did the change follow mania, medication or substance exposure, or endocrine symptoms? The answers determine the first action. For example, behavior confined to personal care and responsive to redirection supports structured privacy, staff-consistent cueing, and environmental adjustment before medication, as reflected in Table 4.

A ward team can document antecedent, behavior, context, redirectability, and impact; match the least restrictive intervention to the leading mechanism; and prespecify review and de-escalation points. Improvement after environmental structure or treatment of mania informs formulation but does not prove anatomical causation. Non-response should prompt reconsideration rather than automatic escalation, preserving safety, dignity, and consensual intimacy. Behavioral redirection, environmental modifications, and caregiver psychoeducation should each have an explicit role in the care plan: a consistent neutral cue and alternative activity, correction of privacy or overstimulation triggers, and coaching that reduces punitive or inconsistent responses. These components should be reassessed alongside any drug treatment, using redirectability, participation, distress, and preserved consensual intimacy as outcomes. They remain active treatment when medication is indicated, rather than preliminary steps abandoned after prescribing.

Patient-centered care must address dysregulation alongside the more common loss of sexual function or intimacy. Education, partner communication, privacy, and rehabilitation goals remain appropriate when capacity is preserved; severe anosognosia or acute mania may require temporary controls or syndrome-targeted treatment. Published anatomically vivid cases are disproportionately male, and observer gender, relationship role, ward culture, and legal exposure may influence reporting. Current evidence cannot establish sex-specific incidence or lesion effects.

### 8.2. Clinical Interpretation and Patient-Centered Care

Precise phenotyping remains the most immediate improvement available to practice and research. Increased desire and impaired control can coexist, but either may occur without the other. These presentations require different treatment targets. Constructs from compulsive sexual behavior and behavioral addiction can inform terminology [177,178,179,180]. Application to ABI must account for impaired self-report and the opportunity for behavioral expression.

Mechanism-based formulation should identify a modifiable difficulty and specify how improvement will be recognized [181,182,183,184,185,186,187,188,189,190,191,192]. If impaired stopping is suspected, response to a consistent cue is more informative than a lesion label. Patient accounts establish subjective experience; collateral observations describe circumstances the patient may not recall. Neither replaces decision-specific capacity assessment.

### 8.3. Research Priorities and Limitations

Prospective multicenter cohorts should preregister phenotype definitions and distinguish subjective desire from observed behavior, compulsivity, disinhibition, and atypical interest. Registries should enroll consecutive eligible patients where feasible, link the clinical timeline to lesion characterization, and record the setting and source of each behavioral observation, including intervals with no target behavior and intervals without adequate observation. This helps distinguish persistent change from altered observation opportunities. Capacity, empathy, moral-emotional judgment, and risk measures should inform but not replace case-specific reasoning [193,194,195,196,197,198,199,200,201,202].

Imaging studies should connect lesion or connectivity measures to standardized neuropsychological phenotyping and repeated real-world sexual-regulation outcomes [203,204,205,206,207,208,209,210,211,212,213,214,215,216]. In TBI, analyses must distinguish the effects of injury severity and chronicity from the contribution of focal contusions or diffuse damage. Stroke analyses should test whether lesion location adds information beyond lesion burden and clinical context. Recent imaging studies demonstrate why external validation and phenotype specificity are essential [6,98,112,113].

Rare-phenotype research should use the least intrusive ascertainment capable of answering the question. Private, accessible interviews and predefined event logs are preferable to unnecessary intimate narratives, photographs, or continuous audio/video recording. Research participation and disclosure of sexual-history information require a separate consent process from routine care; communication support, an appropriately authorized representative when applicable, attention to assent or dissent, and reassessment when capacity changes should follow the approved protocol. Declining an optional sexual-history item should not jeopardize rehabilitation. Participant and caregiver input can help select acceptable questions and meaningful outcomes [217].

A core dataset should use coded behavioral categories, observation opportunities, coarse contextual variables, and relative timing, while storing linkage identifiers separately with restricted access. Rare combinations of lesion images, occupation, exact dates, locations, and intimate case details can permit re-identification even after names are removed. Multicenter protocols should therefore specify access roles, retention and secondary-use permissions, protected data transfer, and disclosure review before releasing small cells, narratives, or imaging; controlled access may be more appropriate than unrestricted participant-level release. Research documentation must remain distinct from clinical safeguarding records and should not collect identifying details about third parties unless necessary. These safeguards complement the minimum fields in Supplementary Table S7.

Intervention research should describe the treatment components and co-interventions clearly enough to interpret benefit and harm. A reduction in incidents should be considered alongside adverse effects, participation, and preservation of appropriate intimacy. Digital or wearable measurement should not default to continuous surveillance of intimate activity; any such method requires a separate justification of necessity, consent, proportionality, and privacy protections.

Important limitations remain. The 13-publication case register improves traceability but does not make this concept-driven narrative search exhaustive or support pooled prevalence. English-language eligibility and full-text availability may have excluded informative reports and underrepresented cultural or care settings. Case descriptions differ in premorbid detail and follow-up, and some patients may appear in more than one publication. Recent guidance supports proactive, partner-inclusive sexuality care [13,21,22,74,75,76,77,78,79,80,81,82,100,101,102,103,104], but mainly addresses sexual function or service delivery. Anatomical interpretations also vary in certainty, from demonstrated lesions to clinically inferred localization.

Comparative designs should quantify opportunity to observe behavior across intensive care, rehabilitation, home, and community settings. Repeated assessment is preferable because awareness and supervision change during recovery, as do medication exposure and relationships.

A minimum dataset should connect injury characteristics to the premorbid baseline and a time-resolved behavioral description. Clinical context must permit evaluation of alternative explanations, with capacity and reporting source documented. Supplementary Table S7 outlines core fields. Consistent reporting and follow-up would be more informative than anatomically striking but clinically incomplete narratives.

## 9. Conclusions

Post-ABI sexual dysregulation is heterogeneous, and interpretation depends on phenotype and clinical course. This review connects those observations to appropriately limited anatomical and treatment inferences. TBI can reveal distributed vulnerability; stroke can identify candidate network nodes. Neither determines sexual preference, consent capacity, or responsibility, which require evidence about the individual’s behavior and understanding. The relative contributions of desire, salience, and executive control require individual assessment.

Clinical care should begin with observable behavior and a testable differential formulation, followed by decision-specific capacity assessment and proportionate safeguarding. Reassessment after treatment of a contributing syndrome helps avoid unnecessary restriction. Behavioral redirection, environmental modification, and caregiver psychoeducation are active therapeutic components and should usually precede, and continue alongside, any indicated pharmacotherapy. Each component requires a defined target and monitoring of benefit, harm, participation, and possible withdrawal. Progress requires prospective cohorts that measure the same phenotype repeatedly and connect anatomical findings to everyday outcomes. Safety and preservation of consensual intimacy should remain concurrent goals. The resulting framework is a clinically usable map of uncertainty: it identifies what is observed, which network mechanisms are plausible, and what the evidence cannot determine. Its value lies in guiding proportionate care and testable hypotheses, not in asserting deterministic localization or inferring a person’s preference, intent, or consent capacity from an image.

## Figures and Tables

**Table 1:** Neurobiological substrates of human sexual behavior and their putative relevance to post-acquired brain injury sexual dysregulation. This table summarizes brain regions and networks relevant to sexual regulation and outlines how injury may contribute to dysregulated phenotypes in a non-deterministic framework.

Substrate or Network	Core Regulatory Contribution	Putative Dysregulatory Consequence When Affected	Possible Clinical Relevance	Evidence Basis and Interpretive Caution
Orbitofrontal cortex (OFC)	Stimulus-value updating, reversal learning, flexible social rule application.	Reduced contextual updating and weaker inhibition of responses that are no longer appropriate.	Sexual comments, intrusive behavior, or impaired boundary awareness when combined with contextual or executive failure.	Direct post-ABI: selected frontal/temporal cases, without an isolated OFC effect [19]. Mechanistic extrapolation: reward/value and reversal-learning models [39,40]; orbitofrontal tumor compression is a neurological comparator [20].
Ventromedial prefrontal cortex (vmPFC)	Affective valuation, somatic integration, consequence evaluation, and moral-emotional reasoning.	Knowledge–action dissociation and reduced emotional weighting of social rules.	Behavior may persist despite verbal knowledge that it is inappropriate.	Direct post-ABI sexual evidence: no selective vmPFC effect established in the cited sources. Mechanistic extrapolation: lesion-based decision-making and valuation models [1,2,41]; impaired judgment does not establish a sexual phenotype.
Anterior cingulate cortex (ACC)	Conflict monitoring, error detection, motivation, and effortful control.	Reduced detection of conflict between desire, impulse, and social constraint.	Persistence despite correction, discomfort of others, or contextual warning cues.	Direct post-ABI sexual evidence: no isolated ACC effect established. Mechanistic extrapolation: conflict-control models [42,59,60,61]; post-TBI corticostriatal imaging assessed executive function, not sexual outcomes [6].
Amygdala	Detection of biologically and socially salient cues and affective significance.	Altered salience attribution or reduced sensitivity to distress-related cues.	Approach behavior or excessive cue salience when combined with impaired top-down regulation.	Direct post-ABI: mixed temporal-injury descriptions do not isolate an amygdala effect [27]. Mechanistic extrapolation: salience and emotion-processing evidence [44,45]; this does not establish a substrate for a particular sexual preference.
Hippocampus	Contextual memory, autobiographical meaning, and relational embedding of experience.	Reduced contextualization of behavior within place, relationship, or prior experience.	Behavior may be poorly matched to setting, relational history, or privacy context.	Direct post-ABI: temporal-injury reports do not isolate a hippocampal sexual-behavior effect [27]. Mechanistic extrapolation: memory lesion-network evidence [69] supports contextual-memory reasoning, not a demonstrated sexual-dysregulation pathway.
Hypothalamus	Endocrine, autonomic, and physiological components of arousal.	Possible disruption of arousal, endocrine-autonomic regulation, or hormonal modulation.	Arousal dysregulation may become clinically relevant when control systems are also impaired.	Direct post-ABI: sparse, anatomically mixed diencephalic observations [14]. Mechanistic extrapolation: endocrine-autonomic and hormonal models [46,58]; tumor compression is an indirect comparator [30]. Hormones or localization alone do not explain complex behavior.
Anterior insula	Interoception, subjective bodily awareness, and integration of internal state with salience.	Altered awareness of bodily arousal or internal escalation.	Poor recognition of internal triggers, arousal intensity, or need for self-regulation.	Direct post-ABI sexual evidence: no selective anterior-insula effect established in the cited sources. Mechanistic extrapolation: interoception and bodily awareness models [43], without direct validation of a sexual-dysregulation mechanism.
Mesolimbic reward system	Motivation, reinforcement learning, incentive salience, and approach behavior.	Increased cue-driven reward seeking or repetitive approach behavior.	Compulsive or repetitive sexual behavior when reward bias coexists with impaired inhibition.	Direct post-ABI sexual evidence: no selective mesolimbic effect established. Mechanistic extrapolation: reward, incentive-salience, and addiction models [37,38,47,48], with non-ABI sexual-cue imaging [5]; neither predicts a post-injury preference.
Frontostriatal circuits	Action selection, response inhibition, habit control, and top-down regulation.	Impulsivity, impaired stopping, habit-driven repetition, or risk-taking.	Repetitive sexual behavior, risk-taking decisions, or difficulty stopping after feedback.	Direct post-ABI sexual evidence: no circuit-specific relationship established. Mechanistic extrapolation: executive-control models [59,60,61] and post-TBI corticostriatal imaging [6]; the latter is direct for cognitive outcomes, indirect for sexual dysregulation.
Prefrontal–limbic and social–cognitive balance	Integration of inhibition, affective salience, reward, social cognition, and contextual judgment.	Network imbalance after focal or diffuse injury.	Cross-etiology vulnerability to sexual dysregulation when multiple regulatory components fail.	Direct post-ABI: clinical co-occurrence of inappropriate sexual and other challenging behaviors [18], without network localization. Mechanistic extrapolation: executive and social–cognitive models [59,60,61,62,63] and non-ABI sexual-cue imaging [5]; this remains a conditional network account.

Abbreviations: ABI, acquired brain injury; ACC, anterior cingulate cortex; OFC, orbitofrontal cortex; vmPFC, ventromedial prefrontal cortex. Note: Direct post-ABI evidence in this table means an observed sexual-dysregulation outcome after TBI or stroke, not simply a study conducted in an injured population. Each row distinguishes this evidence from mechanistic extrapolation; ‘not established’ refers to the cited evidence and is not proof that an association cannot exist. The proposed relationships remain mechanistic and interpretive. They do not imply that any single region determines sexual behavior, sexual identity, or sexual preference, and they do not establish a person’s awareness of legal responsibility or answer questions reserved for legal evaluation.

**Table 2:** Comparative neurobiological model of sexual dysregulation after traumatic brain injury and stroke. This table contrasts traumatic brain injury and stroke as complementary, non-deterministic models of post-acquired brain injury sexual dysregulation.

Feature	Traumatic Brain Injury	Stroke	Evidence Basis and Main Interpretive Limitation
Lesion pattern	Diffuse or multifocal injury, often involving DAI, frontal or temporal contusions, and disconnection.	Focal or strategic vascular lesions with possible remote network effects or diaschisis.	TBI evidence supports distributed network injury [83,84,85,86,87,88,95,96,97]. Stroke evidence supports focal and subcortical behavioral syndromes, but the published case literature is vulnerable to selection and publication bias [28,105,106,107,108,109,110,111,114,131,135].
Main inferential strength	Network-level vulnerability and behavioral dyscontrol across real-world settings.	Lesion-symptom and lesion-network localization of strategic nodes.	TBI is often less anatomically specific. Stroke can be more anatomically informative, while still remaining non-deterministic [69,128,129,130,131,132,133,134].
Candidate mechanisms	Frontostriatal and frontolimbic disconnection, impaired self-monitoring, reduced inhibition, and social-behavioral dyscontrol.	Frontal, temporal-limbic, basal-ganglia, insular, or diencephalic node disruption, including thalamic and hypothalamic structures, within distributed networks.	Mechanisms are inferred from neurobehavioral, imaging, and lesion-network evidence because imaging studies rarely include sexual dysregulation as a prespecified outcome [28,87,88,109,110,132,133,134].
Hypersexuality	Possible, usually interpreted within broader dysexecutive, mood, medication, or rehabilitation-context factors.	Possible but rare, sometimes highlighted by lesion-symptom association with focal or strategic lesion timing.	Do not interpret as lesion-specific causation. Definitions vary and confounders are common [14,17,71,118].
Sexual disinhibition	Linked to behavioral dyscontrol, impaired insight, poor cue processing, and demands of the rehabilitation environment.	Possible, often anatomically informative when frontal or strategic nodes are involved.	Observed behavior may not imply increased libido. Staff/caregiver reporting may influence interpretation [18,71,93].
Acquired paraphilic manifestations	Rare, heterogeneous, and difficult to separate from disinhibition, impulsivity, or expression of pre-existing interests.	Rare, with focal lesion reports potentially informative but vulnerable to publication bias and incomplete premorbid data.	Use “manifestations” cautiously. Do not equate ABI with paraphilic disorder, criminality, or sexual offending [30,118,119,120,121,122,123,124,125,136,137].
Population and context	Often younger cohorts with active social roles, rehabilitation exposure, and long-term community reintegration challenges.	Often older cohorts with vascular burden, medical comorbidity, aphasia, neglect, or mood complications.	Population differences affect reporting, opportunity, caregiver burden, and generalizability [105,106,107,108,131,135].
Main limitation	Heterogeneity and diffuse injury limit localization.	Rarity, publication bias, diaschisis, and comorbidity limit causal certainty.	Both etiologies require multilevel formulation integrating lesion anatomy, cognition, mood, medication, context, and collateral reports [69,133,134].

Abbreviations: ABI, acquired brain injury; DAI, diffuse axonal injury; TBI, traumatic brain injury. Note: TBI and stroke are presented as complementary inferential models, not as deterministic etiological categories. Both can involve focal and distributed network effects.

**Table 3:** Clinical phenotypes of post-acquired brain injury sexual dysregulation. This table separates overlapping behavioral phenotypes to support precise clinical description and safer interpretation.

Phenotype	Operational Description	Mechanistic Hypotheses	Key Differential Considerations	Clinical Caution and Representative Sources
Increased libido	Subjectively increased desire, initiation, or sexual interest.	Hormonal modulation, mood elevation, medication exposure, or reward sensitivity.	May coexist with erectile, orgasmic, fatigue-related, or relationship-related sexual dysfunction.	Clarify desire separately from behavior and opportunity. Hormones alone do not explain complex behavior [11,12,58,105].
Hypersexuality	Excessive preoccupation, urges, initiation, frequency, or sexual activity relative to baseline and context.	Reward salience, mood elevation, disinhibition, compulsive features, or impulse-control mechanisms.	Distinguish from sexual disinhibition, mania/hypomania, medication effects, and environmental opportunity.	Definitions vary. Avoid assuming a new sexual preference or paraphilic disorder [14,17,118].
Sexual disinhibition	Failure to inhibit sexual speech or behavior when context, privacy, consent, or boundaries make expression inappropriate.	OFC/vmPFC dysfunction, frontostriatal impairment, reduced self-monitoring, poor social cue interpretation.	May occur without increased libido. Consider aphasia, anosognosia, delirium, mania, and ward context.	Describe observable behavior before assigning diagnostic labels [18,71,93,135].
Compulsive sexual behavior	Repetitive sexual behavior experienced as difficult to control or associated with repetitive urges.	Habit circuits, reward learning, anxiety relief, compulsivity, or impaired inhibitory control.	Differentiate from frontal perseveration, boredom, impaired privacy awareness, or reduced environmental structure.	Compulsive sexual behavior disorder (CSBD) constructs may inform terminology but should not be imported wholesale into ABI populations [5,120,138].
Context-inappropriate sexual behavior	Sexual comments or acts occurring in settings where they are unsafe, intrusive, non-private, or socially inappropriate.	Poor social cognition, privacy unawareness, reduced insight, impaired contextual judgment.	Evaluate setting, triggers, redirectability, staff/caregiver interpretation, and supervision level.	Highly context-dependent. Avoid moralizing or assuming increased desire [18,71,135].
Risk-taking sexual behavior	Unsafe, impulsive, or poorly considered sexual decisions.	Reward seeking, impaired consequence evaluation, impulsivity, mood elevation, or substance use.	Assess vulnerability, capacity, coercion, consent, substance use, and medication effects.	Risk formulation should be individualized and not reduced to lesion location [59,60,61,127].
Acquired paraphilic manifestations	Rarely reported atypical sexual manifestations temporally associated with brain injury or neurological disease.	Fronto-temporal dysfunction, reward dysregulation, impaired moral-emotional judgment, disinhibition, or expression of pre-existing interests.	Distinguish paraphilic interest, behavior, disorder, unlawful conduct, and sexual offending.	Use cautiously. Do not equate ABI with paraphilic disorder, criminality, or sexual offending [30,118,119,120,121,122,123,124,125,136,137].

Abbreviations: ABI, acquired brain injury; CSBD, compulsive sexual behavior disorder; OFC, orbitofrontal cortex; vmPFC, ventromedial prefrontal cortex.

## Data Availability

No new data were created or analyzed in this study. Data sharing is not applicable to this article.

## Supplementary Materials

The following supporting information can be downloaded at: https://www.mdpi.com/article/10.3390/brainsci16090995/s1, Table S1: SANRA-guided search strategy, screening, eligibility, evidence charting, and narrative-synthesis framework; Table S2: Bibliographic, participant, clinical, and anatomical characteristics of the assessed case reports and case series; Table S3: Clinical timing, treatment, follow-up, outcomes, and limitations of the assessed case reports and case series; Table S4: Neuroanatomical regions and networks implicated in acquired hypersexuality and sexual disinhibition; Table S5: Neurochemical and endocrine pathways potentially involved in post-acquired brain injury sexual dysregulation; Table S6: Reported etiological contexts and clinical presentations of acquired paraphilic manifestations after brain injury; Table S7: Research gaps, recommended design features, and a proposed minimum behavioral observation log; Table S8: Evidence identified through the final 6 August 2026 search and contribution to the review.
