# Supplementary material for: Sexual Dysregulation After Traumatic Brain Injury and Stroke: A Critical Narrative Review of Neurobiology, Clinical Phenotypes, and Management
Source: Brain Sci. 2026 Sep 19;16(9):995. doi: 10.3390/brainsci16090995 (PMC13604643; doi:10.3390/brainsci16090995)
Supplement: Supplementary file 1 [file brainsci-16-00995-s001.zip › brainsci-4538369-supplementary.pdf]

Supplementary Material

Supplementary Table S1. SANRA-guided search strategy, screening, eligibility, evidence charting, and narrative-synthesis framework.

This table operationalizes the methodology reported in the main manuscript, including independent two-reviewer screening, evidence-layer assignment, critical appraisal, and the boundaries of the narrative-review design.

Methodological stage	Operational procedure	Evidence handling and interpretive safeguard
**Review design and SANRA framework**	Structured critical narrative review guided by transparent narrative-synthesis recommendations and the Scale for the Assessment of Narrative Review Articles (SANRA) [23-25]. All six SANRA domains were considered: justification of importance, statement of aims, description of the literature search, referencing, scientific reasoning, and presentation of relevant endpoint data.	SANRA guided reporting and final internal quality assurance. It was not used as a substitute for a systematic-review protocol, a universal risk-of-bias score, or a numerical claim of certainty.
**Review objectives and analytic domains**	The review addressed four linked objectives: phenotype definition; comparison of the inferential contributions of TBI and stroke; anatomically constrained interpretation with separation of direct ABI evidence from extrapolation; and translation into assessment and management. Synthesis domains were phenotype, neuroanatomy/network mechanisms, differential assessment, and management/service delivery.	Sources were retained for a defined contribution to at least one objective rather than for simple topical mention.
**Sources, coverage, and language**	Two authors (R.S.C. and A.C.) searched PubMed/MEDLINE, Scopus, and Web of Science from database inception through 6 August 2026. Eligibility was restricted to English-language publications.	English-language full-text eligibility permitted direct appraisal of clinical details. This restriction may omit informative cases and introduce language, cultural, or geographical bias.
**Concept blocks**	Searches combined ABI-etiology terms (TBI, head injury, acquired brain injury, stroke, cerebrovascular and strategic-lesion terms) with phenotype terms (hypersexuality, sexual disinhibition, inappropriate or compulsive sexual behavior, and acquired paraphilic manifestations). Supplementary searches incorporated anatomy, network, neurochemical, endocrine, assessment, management, rehabilitation, consent, and capacity concepts.	Mechanistic terms were used to identify evidence needed to interpret direct clinical reports; domain-general mechanistic studies were not treated as direct validation of post-ABI sexual dysregulation.
**PubMed/MEDLINE strategy**	(("Brain Injuries, Traumatic"[Mesh] OR "traumatic brain injur*"[tiab] OR "head injur*"[tiab] OR "acquired brain injur*"[tiab] OR "Stroke"[Mesh] OR stroke[tiab] OR cerebrovascular[tiab] OR "frontal lesion*"[tiab] OR "temporal lesion*"[tiab] OR "thalamic infarct*"[tiab]) AND (hypersexual*[tiab] OR "sexual disinhibit*"[tiab] OR "inappropriate sexual behavior*"[tiab] OR "inappropriate sexual behaviour*"[tiab] OR "compulsive sexual behavior*"[tiab] OR "compulsive sexual behaviour*"[tiab] OR paraphil*[tiab] OR "acquired pedophil*"[tiab] OR exhibitionis*[tiab] OR voyeuris*[tiab] OR frotteur*[tiab]))	Additional PubMed searches used the mechanism and clinical-management concepts described above. Both US and UK spellings of behavior were represented explicitly.
**Scopus strategy**	TITLE-ABS-KEY(("traumatic brain injur*" OR "head injur*" OR "acquired brain injur*" OR stroke OR cerebrovascular OR "frontal lesion*" OR "temporal lesion*" OR "thalamic infarct*") AND (hypersexual* OR "sexual disinhibit*" OR "inappropriate sexual behavio?r*" OR "compulsive sexual behavio?r*" OR paraphil* OR "acquired pedophil*" OR exhibitionis* OR voyeuris* OR frotteur*))	Supplementary mechanism terms were applied with TITLE-ABS-KEY fields. Scopus was also used for forward citation checking.
**Web of Science strategy**	TS=(("traumatic brain injur*" OR "head injur*" OR "acquired brain injur*" OR stroke OR cerebrovascular OR "frontal lesion*" OR "temporal lesion*" OR "thalamic infarct*") AND (hypersexual* OR "sexual disinhibit*" OR "inappropriate sexual behavio?r*" OR "compulsive sexual behavio?r*" OR paraphil* OR "acquired pedophil*" OR exhibitionis* OR voyeuris* OR frotteur*))	Supplementary mechanism terms were applied with TS fields. Web of Science was also used for forward citation checking.
**Final evidence update**	The final searches completed on 6 August 2026 sought recent clinical, guideline, implementation, endocrine, cognitive-profile, functional-connectivity, self-awareness, and lesion-network evidence relevant to the existing synthesis.	Updated mechanistic imaging studies were classified as indirect unless sexual dysregulation was an explicitly measured outcome; their contribution is summarized in Supplementary Table S8.

Methodological stage	Operational procedure	Evidence handling and interpretive safeguard
**Record collation and deduplication**	The two search authors (R.S.C. and A.C.) collated retrieved records and removed duplicates before eligibility screening. Citation chaining was a complementary identification stream.	The 13-publication case-report register is provided in Supplementary Tables S2 and S3. This publication inventory is distinct from a database-wide retrieval denominator; no numerical screening flow was retrospectively estimated.
**Backward and forward citation checking**	The same two authors (R.S.C. and A.C.) checked reference lists and citing papers for key clinical studies, landmark lesion reports, rehabilitation observations, measurement sources, guidelines, implementation studies, and treatment reports.	Citation chaining was used to identify terminology variants, landmark evidence, and otherwise difficult-to-retrieve direct reports; it was not used to infer prevalence or completeness.
**Title and abstract screening**	Two reviewers (R.S.C. and A.C.) independently screened titles and abstracts against the eligibility criteria. Any record judged potentially relevant by either reviewer advanced to full-text assessment.	The inclusive advancement rule reduced the chance that a rare, historically described, or differently labelled phenotype would be excluded by one reviewer at the first stage.
**Full-text assessment and disagreements**	The same two reviewers (R.S.C. and A.C.) independently assessed potentially eligible full texts. Decisions considered phenotype, ABI etiology, lesion timing or network mechanism, and contribution to differential diagnosis, measurement, management, or implementation. Disagreements at either stage were resolved through discussion and consensus.	Final eligibility required reviewer consensus; no disagreement was carried forward unresolved.
**Inclusion criteria**	Eligible sources included clinical studies, rehabilitation cohorts, case reports or series, lesion observations, neuroimaging studies, mechanistic reviews, clinical guidelines, implementation studies, and management papers that directly informed post-ABI sexual dysregulation or an explicitly necessary differential mechanism.	Eligibility required an identifiable contribution to phenotype, anatomy/network inference, assessment, differential diagnosis, treatment, safeguarding, or service delivery.
**Exclusion criteria**	Duplicate publications, non-English reports, papers without relevant ABI, phenotype, mechanism, assessment, or management content, and non-ABI sources that did not provide a necessary mechanistic or clinical comparator were excluded. Case-based reports without an available full text were excluded from the case-based synthesis.	Non-ABI evidence was retained only when its indirectness and purpose were made explicit.
**Evidence-layer assignment**	Sources were classified as: (1) direct evidence on post-ABI sexual dysregulation; (2) broader post-ABI sexual-function, rehabilitation-service, guideline, or implementation evidence; or (3) domain-general mechanistic or comparator evidence.	Evidence layers were not pooled. Direct clinical observations, implementation outcomes, and mechanistic plausibility were interpreted separately.
**Retention of older evidence**	Recent studies were preferred for equivalent claims. Older sources were retained when foundational, instrument-validating, anatomically landmark, or the only direct clinical evidence for a specific ABI presentation.	Publication age alone was neither an inclusion nor exclusion criterion; the source's unique evidentiary role was required.
**Case-report and case-series handling**	Supplementary Table S2 reports the bibliographic, participant, clinical, and anatomical characteristics of all 13 English-language case-based publications in the assessed register. Supplementary Table S3 reports clinical timing, interventions, follow-up, outcomes, and limitations.	The register distinguishes direct TBI/stroke observations, mixed neurological sources, and non-ABI pharmacological comparators. The sertraline–hypomania case was retained only for the medication differential. Publication-level sample sizes were not pooled; overlapping cases and variable phenotype ascertainment prevent an aggregate patient estimate. The ten neurological publications are a qualitative set, not ten independent anatomical experiments; the three pharmacological comparators are kept separate from their neurological inference.
**Data charting**	Two authors (R.S.C. and A.C.) undertook charting. For each included source, they charted citation, population and ABI etiology, design, phenotype or outcome, lesion or imaging information, temporal relationship, premorbid characterization, follow-up, evidence layer, contribution to the review question, and principal limitation.	Charting fields were chosen to expose directness, temporality, missing premorbid information, and clinically important confounding rather than to create a pooled effect estimate. For premorbid inference, an unreported history was treated as unknown; retrospective informant descriptions were not equated with independently verified absence of prior interest. The inference could therefore concern newly observed behavior without establishing a de novo preference.
**Critical appraisal and treatment hierarchy**	Two authors (R.S.C. and A.C.) appraised design, ABI directness, phenotypic clarity, anatomical precision, temporality, confounding, sex and gender reporting, and the source of behavioral observation. They additionally graded treatment evidence with the 2011 Oxford Centre for Evidence-Based Medicine hierarchy [34].	Case reports and series remained hypothesis-generating. OCEBM levels describe design and directness of treatment evidence and do not by themselves constitute recommendations.

Methodological stage	Operational procedure	Evidence handling and interpretive safeguard
**Narrative synthesis**	Two authors (R.S.C. and A.C.) synthesized the evidence. Evidence was compared across TBI and stroke, then integrated across inhibition, salience, reward, interoception, self-awareness, social cognition, contextual judgment, clinical differential diagnosis, and management. Contradictions and indirectness were retained rather than averaged away.	The synthesis supports probabilistic regulatory-network models; it does not support a one-lesion-one-behavior or 'sexual center' interpretation. Each case was compared on phenotype, evidence of injury or exposure, anatomical certainty, onset, premorbid information, competing explanations, follow-up, and evidence role. Inconsistencies, spontaneous improvement, normal test findings, and uncertain localization were preserved rather than removed to make the model appear coherent.
**Figure development and MRI provenance**	Conceptual figures were derived from the evidence synthesis and established neurobehavioral literature [35]. De-identified T1-weighted MRI backgrounds were obtained from OpenNeuro dataset ds006072, version 1.1.0 [36].	No participant-level, functional, medication, or outcome data were analyzed. Colored overlays are author-controlled schematics, not atlas-derived or subject-specific segmentations, and do not imply causality. Figure legends explicitly distinguish these conceptual overlays from quantitative lesion maps, statistical maps, and measured patient-level connectivity or behavioral risk.
**Methodological boundary and reporting limitation**	This was a critical narrative review rather than an exhaustive systematic review of prevalence or intervention effects. The case-based register comprises 13 assessed publications. A PRISMA flow diagram and pooled estimate were not applied, and no database-wide retrieval denominator was inferred from the case register.	The review does not claim exhaustive retrieval, pooled prevalence, or quantitative certainty. English-language eligibility, full-text availability, and selective case publication constrain generalizability.
**Qualitative retention of neurological exemplars**	An identifiable phenotype or differential syndrome, a defined neurological context, and an explicit contribution to timing, anatomy, assessment, or management justified retention. Full-text case descriptions were compared using the charted fields, not selected through a SANRA score cutoff.	Ten neurological publications include single cases and series, direct TBI/stroke observations, mixed etiologies, and neurological comparators. They remain distinct from three non-ABI pharmacological comparators; incomplete imaging or history limits inference rather than establishing a clean anatomical model.
**Publication bias and missing premorbid histories**	Striking or legally salient cases were treated as selectively published. Unreported histories were coded as unknown in interpretation; retrospective collateral descriptions were distinguished from independently documented baselines. Newly observed behavior was separated from a newly established enduring preference.	No frequency of published cases was used as an incidence or localization estimate. Medication, mood, supervision, follow-up, and possible overlap constrained inference. No funnel-plot correction or claim that publication bias had been removed was made.
**Supplementary reference checking**	Focused reference checking of foundational lesion evidence, dopaminergic agents, stroke rehabilitation, research ethics, and endocrine contributors was completed on 14 and 17 September 2026 to support mechanistic and clinical interpretation.	These sources complemented the database search completed on 6 August 2026 and were appraised separately from the 13 publications in the case-based synthesis.

Abbreviations: ABI, acquired brain injury; MEDLINE, Medical Literature Analysis and Retrieval System Online; OCEBM, Oxford Centre for Evidence-Based Medicine; SANRA, Scale for the Assessment of Narrative Review Articles; TBI, traumatic brain injury.

Supplementary Table S2. Bibliographic, participant, clinical, and anatomical characteristics of the assessed case reports and case series.

The case-based synthesis comprises 13 English-language publications assessed in full text: ten neurologically informative case reports or case series and three non-ABI pharmacological comparators. The neurological set comprises Miller, Britton, Spinella, Alnemari, Burns and Swerdlow, Eghwrudjakpor and Essien, Lilly, Caro and Jimenez, Mutarelli, and Sartori; the pharmacological comparators are Mendhekar, Raymond, and Bostwick. These are publication categories, not independent patient totals. Direct TBI/stroke reports are distinguished from mixed neurological sources and non-ABI comparators. Sample sizes refer to each publication and are not pooled. Vancouver citation numbers correspond to the reference list in the main manuscript; rows are ordered by reference number. Supplementary Table S3 presents treatment and longitudinal information for the same publications. Premorbid information is reproduced at the level supported by each report. Missing documentation does not establish an absent prior interest or prove a newly acquired preference.

Study	Design and participants	Etiology and clinical context	Sexual phenotype and premorbid information	Anatomy and assessment
Miller et al. (1986) [14]	Descriptive series; 8 patients (6 men, 2 women), ages 30–75 years. Multicenter neurological observations.	Mixed neurological etiologies: aneurysmal hemorrhage, postoperative injury, stroke, postencephalitic epilepsy, glioma, postencephalitic hydrocephalus, herpes encephalitis, and levodopa-treated Parkinson disease. Not a TBI series.	Four hypersexual/disinhibited presentations and four presentations described as altered preference. Premorbid histories came largely from partners. Manifestations ranged from public sexual approaches and postictal arousal to atypical interests and genital self-injury.	Basal-frontal/septal hemorrhage; postoperative bilateral basal-frontal lesions; right thalamic–hypothalamic injury; temporal epileptiform activity; hypothalamic/brainstem glioma; hydrocephalus with a shunt tip near hypothalamic–septal structures; bitemporal dysfunction; normal CT in the Parkinson case. CT, EEG, clinical examination, and selected postmortem findings.
Britton (1998) [15]	Single case; man aged 36 years at evaluation, injured at 21 years. Nursing-home resident assessed in a rehabilitation service.	Severe motorcycle-related TBI with prolonged profound disability. Limited speech, dependent transfers, gastrostomy nutrition, and catheter-based bladder care.	Intrusive sexual touching and prolonged masturbation, accompanied by aggression toward male staff/residents. Aggression began 8 years after injury and the broader pattern progressively intensified. Staff and family supplied collateral information.	MRI: symmetric periventricular white-matter signal abnormalities and moderate diffuse cerebral atrophy. EEG and routine laboratory studies were normal. Simple-command comprehension with markedly restricted verbal communication.

Study	Design and participants	Etiology and clinical context	Sexual phenotype and premorbid information	Anatomy and assessment
Spinella (2004) [17]	Single case; right-handed man aged 66 years. Retired university professor, previously independent; evaluated during acute rehabilitation.	Acute cerebrovascular event with massive intraventricular hemorrhage and right frontal ventriculostomy. A paramedian thalamic syndrome was clinically inferred.	Sexual/scatological comments, exposure, and public masturbation, with apathy, inappropriate joking, anosognosia, and confabulatory amnesia. Family described the disinhibition as unlike his premorbid personality; no concurrent mania was reported.	CT documented ventricular blood; follow-up noncontrast MRI did not demonstrate an infarct. Left thalamic localization was inferred, with right involvement not excluded. Markedly impaired memory and set shifting; Trail Making A/B at 2nd/1st percentiles; Stroop could not be completed; simple digit span was preserved.
Alnemari et al. (2016) [19]	Single case; patient in the early twenties; sex and exact age NR in the case description. Neurosurgical follow-up with a family informant.	Horse-riding TBI with left temporal fracture, epidural hematoma, and frontotemporal contusions; hematoma evacuated by craniotomy.	Family reported increased sexual desire toward children and teenagers at follow-up, alongside irritability, attention problems, and insomnia. Authors interpreted this as unmasking of previously inhibited interest; detailed independent premorbid verification was limited.	Initial CT: left basal-frontal and bilateral temporal contusions. Follow-up CT: left basal-frontal and temporal encephalomalacia. No seizures or overt neurological deficits reported at the behavioral follow-up; formal cognitive or sexual-outcome scales NR.
Burns et al. (2003) [20]	Single case; right-handed man aged 40 years, a schoolteacher. Neurological assessment during psychiatric hospitalization.	Right orbitofrontal compression by an anterior skull-base hemangiopericytoma. A remote minor head injury 16 years earlier had no apparent sequelae; the presenting syndrome was associated with the tumor.	Escalating pornography use and sexual approaches to a child, with intrusive sexual solicitation despite awareness of inappropriateness. Longstanding pornography interest but no reported prior attraction to children. Constructional apraxia, agraphia, gait disturbance, and incontinence.	MRI: anterior cranial-fossa mass displacing right orbitofrontal and distorting dorsolateral prefrontal cortex. Initial MMSE 25/30 with impaired drawing/writing and verbal fluency; several other cognitive functions were preserved. Histology confirmed hemangiopericytoma.
Eghwrudjakpor et al. (2008) [26]	Five hospital cases in Nigeria: four men (24, 43, 29, 27 years) and one woman (33 years).	Four road-traffic injuries and one assault. Admission GCS: case 1, 3; case 2, 3; case 3, 12; case 4, 15; case 5, NR. Severe, moderate, and mild presentations were represented.	Case 1: sexual demands and grabbing during recovery. Case 2: repeated public masturbation. Case 3: breast exposure, intrusive contact, and sexual demands. Case 4: public masturbation after discharge. Case 5: attempted sexual assault during hospitalization.	Case 2: normal brain CT despite right parietal fracture; case 4: depressed right frontal fracture on radiography. Other cases had no bony injury on skull radiographs. CT/MRI lesion maps were not provided for all patients; case 5 had right spastic paresis.

Study	Design and participants	Etiology and clinical context	Sexual phenotype and premorbid information	Anatomy and assessment
Lilly et al. (1983) [27]	Series of 12 patients; four described in detail. Complete sample-level age/sex distribution NR. Detailed cases included two women and two men.	Klüver–Bucy syndrome: 2 trauma cases, 4 encephalitis cases, 5 Pick disease cases, and 1 Alzheimer disease case (source table). Eligibility required at least three simultaneous syndrome features.	Altered sexual activity was recorded in 7/12 patients, not all 12. Other features included hyperorality, altered eating, placidity, and visual-recognition difficulties, with aphasia, amnesia, or dementia. Detailed TBI case: 57-year-old man with indiscriminate sexual advances.	Bilateral temporal dysfunction supported by variable clinical, operative, imaging, EEG, or pathological evidence. In the detailed TBI case, surgery identified severe bilateral inferior-temporal damage. Neurodegenerative etiologies were confirmed at autopsy in two detailed cases.
Caro et al. (2016) [28]	Two original cases plus review: woman in her fifties; man in his late twenties.	Frontotemporal dementia; MS with epilepsy and previous football concussions. Mixed neurological comparators.	Partial KBS with intrusive touching, hyperorality/hyperphagia, and cognitive/behavioral change. Woman: 15-month decline, MoCA 23/30.	Woman: bilateral hippocampal/right amygdala atrophy and frontotemporal hypometabolism. Man: demyelinating lesions; video-EEG linked symptoms to postictal states.
Mutarelli et al. (2006) [29]	Single case; right-handed man aged 63 years, followed in neurology in São Paulo, Brazil. English full text with a Portuguese abstract.	Bilateral paramedian thalamic ischemic stroke. History of hypertension, myocardial infarction, and coronary bypass; premorbid behavior described as circumspect.	Repeated requests for intercourse with his wife and approaches to other women, with disinhibition, irritability, hypersomnia, and anterograde amnesia. No reported change in sexual preference.	MRI confirmed bilateral paramedian thalamic infarction; SPECT showed bilateral frontal hypoperfusion. Wechsler memory score 52; Wisconsin Card Sorting, language, motor-programming, and constructional testing were reported as normal.
Sartori et al. (2016) [30]	Single case reported in a letter; man aged 64 years, a pediatrician. Neurological/psychiatric assessment during legal proceedings.	Clival chordoma compressing orbitofrontal cortex and hypothalamus, with pituitary displacement and optic-chiasm involvement. No reported premorbid relevant neurological/psychiatric disorder or atypical sexual behavior.	Sexually inappropriate behavior toward a child, with poor appreciation of consequences. Wife described preceding irritability and disinhibition. Pathological crying, compulsive/childish behavior, and impaired emotion attribution, moral reasoning, and abstraction.	MRI: approximately 4 × 3 cm clival tumor. Diplopia/tunnel vision and asymmetric brisk reflexes accompanied frontal behavioral findings. Detailed neuropsychological scores were referenced in the article's separate supplementary material.
Mendhekar et al. (2003) [31]	Single case; woman aged 23 years evaluated in psychiatry in New Delhi, India.	Dissociative disorder with situational anxiety. No reported head injury, seizure disorder, medical/neurological illness, past mood disorder, or family psychiatric history.	After sertraline: increased activity and sociability, reduced sleep, racing thoughts, grandiosity, and elevated affect. Hypomania was the reported outcome; sexual dysregulation was not assessed.	No structural lesion reported. Neurological consultation did not advise neuroimaging. Biochemical investigations, including thyroid tests, were normal.

Study	Design and participants	Etiology and clinical context	Sexual phenotype and premorbid information	Anatomy and assessment
Raymond et al. (2002) [32]	Two treatment cases: woman aged 42 years and man aged 62 years. Outpatient psychiatric/sexual-health setting.	Non-ABI compulsive sexual behavior. Woman: major depression and recent cocaine abstinence; sexual symptoms predated cocaine use. Man: approximately 20-year intermittent history with prior dysthymic symptoms.	Distressing, poorly controlled extramarital sexual behavior. Woman reported persistent urges despite improved depression; man reported intrusive masochistic fantasies and recurrent sexual preoccupation. Bipolar disorder was excluded clinically in the woman.	No ABI or brain lesion reported. Clinical psychiatric assessment; man's Beck Depression Inventory score was 4 at evaluation. No sexual-behavior imaging biomarker or standardized serial sexual scale reported.
Bostwick et al. (2008) [33]	Single case; man first presenting at age 24 years, followed through a recurrent treatment course over subsequent years.	Non-ABI compulsive Internet sexual behavior with depression and episodic binge drinking. Longstanding pornography interest from childhood/adolescence.	Up to 8 hours/day online, prolonged masturbation, repeated loss of employment, relationship disruption, and occasional unprotected encounters. Symptoms caused substantial functional impairment beyond moral distress alone.	No brain injury or lesion was reported. The reward-circuit figure is a mechanistic illustration, not patient imaging. Outcomes were based on clinical follow-up and self-report.

Abbreviations: ABI, acquired brain injury; CT, computed tomography; EEG, electroencephalography; GCS, Glasgow Coma Scale; KBS, Klüver–Bucy syndrome; MMSE, Mini-Mental State Examination; MoCA, Montreal Cognitive Assessment; MRI, magnetic resonance imaging; MS, multiple sclerosis; NR, not reported in the reviewed full text; SPECT, single-photon emission computed tomography; TBI, traumatic brain injury. Sex and age are reproduced as reported.

Supplementary Table S3. Clinical timing, treatment, follow-up, outcomes, and limitations of the assessed case reports and case series.

This table complements Supplementary Table S2 and uses the same publication order. Treatments and doses describe historical case management, not prescribing recommendations. Improvement during recovery, after surgery, or during combination treatment cannot establish an isolated intervention effect. No aggregate patient count or pooled treatment estimate is calculated. Unreported premorbid histories and follow-up were not imputed. The limitations column constrains the claim supported by each report; it does not provide a quantitative correction for selective publication.

Study	Clinical timing and follow-up	Intervention and reported dose	Observed course / outcomes	Evidence role and principal limitations
Miller et al. (1986) [14]	Variable latency. One stroke-associated presentation resolved after approximately 1 month; postictal arousal usually lasted <10 minutes, with one 12-hour episode. Postencephalitic behavioral change persisted for 13 years in one woman. The Parkinson case had 6 years of follow-up.	Case-specific treatment: surgery for vascular/tumor disease; thiothixene with benztropine for the postoperative manic presentation; reduction of levodopa–carbidopa in the Parkinson case. One patient declined shunt revision. Sexual-symptom drug doses were not consistently reported.	Aneurysmal hemorrhage case died 2 weeks after admission. Postoperative manic behavior improved during a 1-month admission, then follow-up was lost. Thalamic-stroke symptoms subsided after 1 month. Glioma case deteriorated and died. Parkinson-associated atypical behavior abated after medication reduction without reported recurrence over 6 years.	Mixed vascular, infectious, neoplastic, postoperative, and medication-related observations; heterogeneous ascertainment. No standardized sexual outcome. The postencephalitic woman appears to overlap with the detailed case in Lilly et al.; do not pool publication-level samples.
Britton (1998) [15]	Approximately 15 years after injury at treatment assessment. Ten-day initial admission; relapse documented 1 month after discharge. Medication response assessed at 1 and 2 months, then monthly and quarterly; final total follow-up duration NR.	Operant behavioral program reduced touching during admission, but was not implemented consistently after discharge. Valproate and several earlier psychotropics were unsuccessful. Medroxyprogesterone acetate 300 mg intramuscularly weekly was then prescribed with guardian approval.	Touching fell from 4–5 to 1–2 episodes/day during inpatient behavioral treatment, then relapsed. After medroxyprogesterone, improvement began within 10 days and control was judged adequate by 3 weeks; touching was reported at 4–5 episodes/week. Masturbation and aggression decreased. No adverse effects were reported; blood counts/metabolic tests remained normal.	Uncontrolled single-patient treatment sequence; differences between inpatient and nursing-home observations and poor behavioral-program implementation confound attribution. Persistent aggression was not eliminated. No dose-reduction trial or defined final follow-up.
Spinella (2004) [17]	Behavioral and cognitive findings documented in the post-acute rehabilitation period. CT at 14 days retained blood in the left posterior lateral ventricle. Longer-term sexual-behavior follow-up NR.	Ventriculostomy for the acute intracranial condition and inpatient rehabilitation. No targeted sexual-behavior treatment or dose was reported.	Detailed cross-sectional behavioral/neuropsychological characterization; no quantified sexual-treatment response or documented long-term remission.	The title refers to thalamic infarction, but the lesion was not demonstrated on follow-up MRI. Localization rests on the clinical syndrome and hemorrhage distribution; ventricular hemorrhage and hydrocephalus-related effects limit focal inference.

Study	Clinical timing and follow-up	Intervention and reported dose	Observed course / outcomes	Evidence role and principal limitations
Alnemari et al. (2016) [19]	Behavioral change documented approximately 3 months after injury/surgery. Subsequent longitudinal sexual-behavior assessment NR.	Craniotomy and epidural hematoma evacuation. No specific intervention for the sexual symptoms, medication dose, or behavioral-treatment protocol was reported.	Good general neurological recovery with persistent reported attention, sleep, irritability, and sexual-behavior concerns at the documented visit. No treatment-response or remission data for sexual symptoms.	Family-reported change, short follow-up, and incomplete premorbid characterization. Does not establish creation of a new preference or a preference-specific anatomical mechanism.
Burns and Swerdlow (2003) [20]	Behavior escalated during 2000. Initial resection in December 2000; return home at 7 months. Headache and pornography collection recurred in October 2001 with tumor regrowth; repeat resection in February 2002.	Before tumor diagnosis: medroxyprogesterone acetate 10 mg/day, fluoxetine, and a sexual-addiction rehabilitation program without adequate control. Tumor resection, subsequent psychosocial rehabilitation, and re-resection after recurrence.	Behavioral and constructional deficits improved after resection; symptoms recurred with regrowth and improved again after repeat surgery. Two days after re-resection, MMSE was 30/30, drawing/writing normalized, and verbal fluency improved.	Neurological comparator with neoplastic etiology, not direct TBI/stroke evidence. Within-person recurrence strengthens temporal association, but tumor mass effect and co-treatment preclude a preference-specific localization or general treatment inference.
Eghwrudjakpor and Essien (2008) [26]	Behavior emerged around week 12 (case 1), after consciousness returned at week 3 (case 2), around week 4 (case 3), at discharge after 2 weeks (case 4), and around week 6 (case 5). Case 4 was lost to follow-up after one outpatient visit.	No specific sexual-symptom drug in any case. Case 1 received an unspecified minor tranquilizer for agitation; case 3 received verbal correction; case 5 received verbal correction and later chlorpromazine for persistent aggression (dose NR). Routine injury care continued.	Case 1 normalized within 2 weeks of behavioral onset; case 2 stopped masturbating publicly by the third week of observation. Case 3's exposure/language subsided. Case 4's longer-term outcome was unknown. Case 5 did not repeat the reported assault, although aggression required treatment.	Uncontrolled recovery-phase observations; spontaneous recovery, supervision, redirection, and sedation cannot be separated. Limited neuroimaging/premorbid data; seizures in case 2 and marijuana history in case 4. Reported verbal correction is historical management, not an endorsed protocol.
Lilly et al. (1983) [27]	TBI case: syndrome apparent about 1 month after the accident and improved during the next month. One postencephalitic woman remained severely impaired 13 years after onset. Dementia cases had progressive courses; uniform follow-up for all 12 NR.	Detailed trauma case underwent evacuation of bilateral blood collections and supportive care. In the postencephalitic woman, phenytoin controlled seizures but haloperidol did not improve the general behavioral syndrome. No standardized sexual-treatment protocol across cases.	The detailed TBI patient stopped inappropriate sexual approaches as language/recognition improved, but could not return to work. Postencephalitic KBS persisted; degenerative cases deteriorated. Findings support variable course by etiology, not a uniform treatment response.	Mixed etiologies and uneven clinical detail; several patients had been described previously. Only two had trauma. Altered sexual activity was one syndrome component and was absent in five cases. Possible overlap with Miller et al. prevents simple patient aggregation.

Study	Clinical timing and follow-up	Intervention and reported dose	Observed course / outcomes	Evidence role and principal limitations
Caro and Jimenez (2016) [28]	Woman: 8-day assessment. Man: readmission 25 days later, lasting 30 days.	Woman: redirection; one 5-mg olanzapine dose; mirtazapine stopped. Man: antiepileptic adjustment and several psychotropics; later seizure/MS treatment.	Woman required institutional care. Man's KBS improved with seizure control and did not recur during readmission.	Two original patients only; review cases are not additional participants. Multiple etiologies/co-treatments prevent TBI-specific or drug-specific inference.
Mutarelli et al. (2006) [29]	Behavioral syndrome became evident over the weeks after stroke. Follow-up extended to 9 years.	Neuroleptics and carbamazepine were tried without benefit for hypersexuality; agents/doses beyond carbamazepine NR. Testosterone-suppressing therapy was not used because of perceived thrombosis risk.	Sexual/behavioral disturbance remained unchanged over 9 years, while memory improved. No controlled treatment response.	Direct stroke case with unusually long follow-up. Normal structured executive testing limits claims of a uniform dysexecutive syndrome; frontal hypoperfusion was associated with, but not proven causal for, the sexual phenotype.
Sartori et al. (2016) [30]	Irritability/frustration reportedly began about 2 years before evaluation. Pre-/postoperative change was described; exact postoperative follow-up duration NR in the reviewed article.	Surgical tumor resection. No separate sexual-symptom pharmacotherapy or dose reported in the reviewed article.	Authors reported regression of behavioral, neurological, and neuropsychological abnormalities, including pedophilic urges, after surgery. Exact serial scores were not reproduced in the reviewed article.	Tumor comparator, not TBI/stroke. Simultaneous compression of several structures limits localization. Postoperative improvement supports temporality but does not establish a universally causal lesion–preference relationship.
Mendhekar et al. (2003) [31]	Hypomanic symptoms began 3–4 days after starting sertraline and were assessed on day 7. Recovery occurred within 7 days after withdrawal; longer-term follow-up NR.	Sertraline 50 mg/day was stopped. Clonazepam 1 mg at night was prescribed during observation; marital counseling followed recovery.	Complete reported resolution of hypomania within 1 week of stopping sertraline. No sexual-outcome measurement or ABI treatment effect.	Retained solely as a medication-related differential/safety comparator. Dechallenge is supportive but co-treatment and absent long-term follow-up limit interpretation; not a sexual-dysregulation case.

Study	Clinical timing and follow-up	Intervention and reported dose	Observed course / outcomes	Evidence role and principal limitations
Raymond et al. (2002) [32]	Woman: improvement within 2 weeks of escalation to 100 mg/day; later recurrence during an antidepressant switch. Good results on final combination for 1 year. Man: initial improvement at 1 month; recurrence at 2 months, then 8 months without recurrence on increased dose.	Woman: naltrexone 50 mg nightly, then 100 mg/day and later 150 mg/day; concurrent fluoxetine, transient citalopram, then fluoxetine 10 mg/day. Man: naltrexone 50 then 100 mg/day with citalopram 40 mg/day. Woman also received individual/group therapy.	Both reported reduced urges/behavior and improved psychosocial functioning. Woman's urges to use cocaine also subsided. No liver-enzyme elevations were reported. Antidepressant-related sedation/sexual side effects prompted medication changes.	Indirect pharmacological evidence. Both received serotonin reuptake inhibitors and lacked blinded/comparative assessment; mood, cocaine history, psychotherapy, and medication changes confound attribution. Historical doses describe these reports and are not dosing recommendations.
Bostwick and Bucci (2008) [33]	Repeated engagement/disengagement from treatment over 7 years. Initial naltrexone benefit within 1 week; combined sertraline/naltrexone follow-up exceeded 3 years.	Sertraline 100 mg/day initially improved mood but did not sustain sexual-symptom control. Oral naltrexone started at 50 mg/day and increased to 150 mg/day. Self-tapering to 25 mg/day was associated with relapse; returning to 50 mg/day restored reported control.	Near-complete sustained remission of compulsive Internet use and depression on combined treatment, with occasional lapses. No alcohol use for 3 years and stable employment for >2 years reported; marriage continued with residual dissatisfaction. Systematic adverse-effect data NR.	Non-ABI, uncontrolled combination treatment with self-reported outcomes. Dechallenge/rechallenge supports within-person association but cannot isolate naltrexone's effect or establish applicability to acquired brain injury.

Abbreviations: ABI, acquired brain injury; CT, computed tomography; EEG, electroencephalography; KBS, Klüver–Bucy syndrome; MMSE, Mini-Mental State Examination; MRI, magnetic resonance imaging; MS, multiple sclerosis; NR, not reported in the reviewed full text; TBI, traumatic brain injury. Outcome descriptions are source-specific observations, not standardized response rates. Non-ABI treatment cases and the hypomania safety comparator are not direct evidence of treatment benefit for post-TBI or post-stroke sexual dysregulation.

Supplementary Table S4. Neuroanatomical regions and networks implicated in acquired hypersexuality and sexual disinhibition.

This table provides an anatomical synthesis of regions and networks discussed in the review. The entries distinguish observations with a post-ABI sexual outcome from mechanistic or comparator evidence. Direct evidence concerning cognition or arousal alone is not direct evidence of sexual dysregulation; none of the entries establishes a deterministic lesion-behavior mapping.

Region or network	Evidence context	Possible mechanistic relevance	Key interpretive caution	Representative sources
Orbitofrontal cortex	Reward/value, reversal-learning, social-behavior, tumor, TBI, and stroke literature.	May support contextual inhibition, stimulus-value updating, and suppression of responses that are no longer appropriate.	Direct post-ABI: selected frontal/temporal cases, without isolated OFC attribution. Indirect: reward/value models and tumor compression; no sexual center is established.	[19,20,39,40]

Region or network	Evidence context	Possible mechanistic relevance	Key interpretive caution	Representative sources
Ventromedial prefrontal cortex	Decision-making, somatic-marker, and moral-emotional judgment literature.	May link affective valuation, consequence evaluation, and knowledge-action integration.	Direct post-ABI sexual evidence: no selective vmPFC effect established. Indirect: decision-making and valuation evidence; this does not identify a sexual phenotype.	[2,41]
Anterior cingulate cortex	Conflict monitoring, cognitive control, and effortful regulation literature.	May contribute to detection of conflict between impulse, desire, and social constraint.	Direct post-ABI sexual evidence: no isolated ACC effect established. Indirect: conflict-control models and executive-function imaging, without a sexual outcome.	[6,42,59-61]
Anterior insula	Interoception and subjective awareness literature.	May support awareness of bodily arousal and internal escalation signals.	Direct post-ABI sexual evidence: no selective insular effect established. Indirect: interoception models; these do not predict a specific behavior.	[43]
Amygdala and hippocampal systems	Emotion, salience, memory, and temporal-limbic models.	May influence salience attribution, threat or distress cue recognition, contextual memory, and relational meaning.	Direct post-ABI: mixed temporal-injury descriptions do not isolate either structure. Indirect: amygdala salience models and memory-network evidence; neither establishes a specific sexual preference or sexual-outcome pathway.	[27,44,45,69]
Hypothalamic and diencephalic regions	Hypothalamic-autonomic models and selected diencephalic lesion observations.	May influence endocrine-autonomic arousal and physiological readiness.	Direct post-ABI: sparse, anatomically mixed diencephalic observations. Indirect: endocrine-autonomic models and tumor comparators; complex sexual behavior cannot be attributed to the hypothalamus alone.	[14,30,46,58]
Mesolimbic reward and ventral striatal systems	Reward, incentive-salience, addiction, and approach-motivation models.	May support reward valuation, reinforcement learning, cue salience, and approach behavior.	Direct post-ABI sexual evidence: no selective mesolimbic effect established. Indirect: reward and addiction models, including non-ABI sexual-cue imaging; sexual-outcome applicability remains unvalidated.	[5,37,38,47,48]
Frontostriatal circuits	Executive control, inhibition, action-selection, and frontal-subcortical circuit literature.	May contribute to response inhibition, habit control, and stopping of prepotent responses.	Direct post-ABI sexual evidence: no circuit-specific effect established. Indirect: inhibition models and post-TBI executive-function imaging; cognitive findings do not establish sexual dysregulation.	[6,59-61]
White-matter tracts and network disconnection	TBI, diffusion imaging, and network dysfunction literature.	Disconnection may impair frontal-limbic, frontostriatal, and long-range control networks even when focal lesions are subtle.	Direct post-ABI evidence here concerns injury and cognition. Extrapolation to sexual dysregulation is indirect because the cited imaging studies did not measure that outcome.	[6,85-88,95-98]

Region or network	Evidence context	Possible mechanistic relevance	Key interpretive caution	Representative sources
Lesion-network and social-cognitive systems	Social cognition, frontotemporal syndrome, and lesion-network literature.	May explain why focal or diffuse injury affects distributed regulatory capacities.	Direct post-ABI sexual evidence is clinical and does not validate a specific network. Indirect: social-cognitive and lesion-network evidence; phenotype-specific prospective validation remains necessary.	[62-64,69,112,113,126-128,133,134]

Abbreviations: ABI, acquired brain injury; OFC, orbitofrontal cortex; TBI, traumatic brain injury.

Supplementary Table S5. Neurochemical and endocrine pathways potentially involved in post-acquired brain injury sexual dysregulation.

This table summarizes modulatory systems that may influence arousal, reward, inhibition, social salience, and endocrine-autonomic state after acquired brain injury.

Pathway	Relevant functions	Possible role after injury	Interpretive caution	Representative sources
Dopamine	Reward learning, motivation, incentive salience, approach behavior, and behavioral activation.	May amplify cue-driven approach or impulse-control symptoms when inhibition is weakened.	Dopamine-agonist-associated impulse-control symptoms are a medication differential supported mainly by Parkinson-disease evidence, not a post-stroke risk estimate. Check levodopa and other catecholaminergic exposures without assuming a uniform class or lesion effect.	[49,50,116,117]
Serotonin	Behavioral inhibition, emotional control, impulse regulation, compulsivity-related mechanisms, and common treatment-emergent sexual dysfunction.	Altered serotonergic modulation may influence disinhibition or compulsive features. SSRIs more commonly reduce sexual function; the cited sertraline case documents hypomania, not a direct post-ABI sexual outcome.	Post-ABI evidence is indirect. Monitor desire, arousal, orgasmic function, agitation, and mood after treatment changes.	[31,51,52]
Noradrenaline	Arousal, vigilance, stress reactivity, and behavioral mobilization.	May increase cue responsiveness or behavioral activation in stimulating contexts.	Effects are context-dependent and do not imply sexual specificity.	[56]
Oxytocin	Attachment, trust, bonding, and social salience.	May shape relational cue processing and the meaning of social approach.	Effects are person- and context-dependent and are not uniformly prosocial.	[57]
Testosterone and sex hormones	Sexual motivation, desire, physiological arousal, and endocrine modulation.	Testosterone replacement can increase desire in hypogonadal men; its effect on post-TBI dysregulation remains unestablished.	Changes in libido require review of treatment timing and dose; increased consensual interest does not establish disinhibition.	[58,67,68]

Pathway	Relevant functions	Possible role after injury	Interpretive caution	Representative sources
Pituitary/endocrine dysfunction after TBI	Pituitary and gonadal regulation, fatigue, mood, libido, and quality of life.	May affect sexual function, fatigue, mood, and arousal after TBI.	Post-TBI hypopituitarism more often reduces desire. Thyroid disease can also alter sexual function; hyperthyroidism does not uniformly increase libido.	[65,66]
Medication-related modulation	Medication effects on libido, arousal, inhibition, agitation, mood, and impulse control.	Review dopamine agonists, levodopa-containing preparations, stimulants, testosterone and other androgens, antidepressants, and other relevant agents. Link initiation, dose escalation, withdrawal, and clinically supervised adjustment to the behavior timeline.	Medication may contribute, unmask vulnerability, or coexist with delirium or mania. A temporal association is not proof; deliberate rechallenge is not required. DARS evaluated motor rehabilitation, not a sexual-behavior effect.	[31,53-55,67,68,116,117,167-169]
Cannabis and cannabinoids	Potential modulation of reward, perception, arousal, and anxiety with marked interindividual variability.	Acute effects may increase or decrease erotic-cue responses; document dose, timing, intoxication, withdrawal, and co-use.	Small non-ABI studies do not establish a post-ABI causal pathway.	[53]
Psychostimulants	Dopaminergic and noradrenergic activation, heightened salience, wakefulness, and behavioral activation.	Intravenous methylphenidate increased reported sexual desire in a small non-ABI study; route and population limit generalization.	Evidence is not ABI-specific; prescribed and non-prescribed exposure must be distinguished.	[54]
Alcohol	Reduced inhibitory control, attentional narrowing, and altered subjective arousal and decision-making.	May increase sexual risk intentions or impair consent-related judgment in selected contexts.	Acute intoxication, chronic use, withdrawal, and caregiver reports require separate assessment.	[55]

Abbreviations: ABI, acquired brain injury; SSRI, selective serotonin reuptake inhibitor; TBI, traumatic brain injury.

Supplementary Table S6. Reported etiological contexts and clinical presentations of acquired paraphilic manifestations after brain injury.

This table summarizes sensitive clinical contexts using cautious, non-deterministic language. The focus is on reported manifestations and plausible mechanisms, not on deterministic causal claims.

Context	Reported manifestation	Plausible mechanism	Critical caution	Representative sources
Orbitofrontal tumor or compression	Pedophilic manifestations or severe sexual disinhibition in selected reports.	Impaired contextual inhibition, valuation, and consequence evaluation.	May not represent a stable new pedophilic interest. A lesion does not establish legal responsibility or lack of responsibility.	[20,30]
Traumatic brain injury	Voyeuristic, exhibitionistic, intrusive, or sexually disinhibited behaviors in selected cases and rehabilitation observations.	Frontal disinhibition, poor self-monitoring, reduced insight, or broader dysexecutive syndrome.	Do not infer criminality, paraphilic disorder, or sexual offending from TBI.	[14,16,18,19,136]

Context	Reported manifestation	Plausible mechanism	Critical caution	Representative sources
Stroke	Late-onset hypersexuality, disinhibition, or atypical sexual behaviors in rare reports.	Strategic lesion effects, lesion-network disruption, mood symptoms, or aphasia/anosognosia-related assessment complexity.	Published case reports are vulnerable to selection and publication bias and often lack premorbid sexual, psychiatric, medication, and longitudinal detail.	[17,105,107,108,114,115]
Temporal-limbic injury or Klüver-Bucy-like syndromes	Hypersexuality, altered salience, approach behavior, or preference-like changes in rare contexts.	Altered emotional valuation, novelty processing, salience, and limbic regulation.	Rare syndromes should not be generalized to most patients with ABI.	[27,28,92]
Frontotemporal dementia comparator	Sexual disinhibition and socially inappropriate behavior in neurodegenerative syndromes.	Loss of empathy, social cognition, frontal regulation, and reward control.	Neurodegeneration is an analogical model and differs from ABI in onset, progression, and mechanism.	[122,126-128]
General paraphilic-interest literature	Population-level paraphilic interests and behavior/disorder distinctions.	Helps distinguish interest, behavior, disorder, distress, harm, and offending.	Do not use neurological cases as simple models for idiopathic paraphilias or sexual offending.	[118-121,123-125]
Forensic and capacity context	Boundary-crossing or unlawful behavior requiring clinical, ethical, or legal assessment.	Impaired inhibition may be relevant to risk formulation, but does not answer legal questions by itself.	Capacity, consent, appreciation, intent, risk, and responsibility are distinct questions.	[156,157,192-201]

Abbreviations: ABI, acquired brain injury; TBI, traumatic brain injury.

Supplementary Table S7. Research gaps, recommended design features, and a proposed minimum behavioral observation log.

This table outlines methodological priorities for future research on post-acquired brain injury sexual dysregulation, with attention to mechanism, real-world validity, privacy, and clinical translation.

Panel A Research gaps and recommended design features

Research gap	Recommended design feature	Expected contribution	Safeguard or implementation caution	Representative sources
Inconsistent terminology	Predefine phenotypes and separate libido, arousal, disinhibition, compulsivity, context-inappropriate behavior, risk-taking, and acquired paraphilic manifestations.	Improves comparability, interpretation, and evidence synthesis.	Definitions should avoid stigmatizing or legally loaded language.	[23-25,118-121,143-145,161,176-178]
Rare and heterogeneous presentations	Develop multicenter registries with consecutive enrollment where feasible, predefined event categories, repeated observation, and explicit recording of no-event and unobserved intervals.	Increases sample size and captures broader clinical variability.	Use coded data and separate linkage keys; restrict access and review small-cell, case-narrative, and imaging disclosures for re-identification risk. Specify retention, secondary use, and protected transfer.	[73,74,207-212]
Weak lesion and network characterization	Combine structural imaging, lesion-symptom mapping, lesion-network mapping, diffusion imaging, and functional connectivity.	Improves mechanistic inference across TBI and stroke.	Imaging must be paired with detailed behavioral phenotyping and cannot replace clinical formulation.	[6,69,95-98,112,113,133,134,202-210]

Research gap	Recommended design feature	Expected contribution	Safeguard or implementation caution	Representative sources
Limited real-world behavioral data	Use patient-, caregiver-, and staff-reported outcomes with structured observation across settings; Panel B specifies proposed minimum fields for a shared log.	Captures behaviors that may not appear in interviews or standard tests.	Record observer role and opportunity for observation; lack of observation is not a zero event count. Avoid unnecessary continuous intimate surveillance or identifiable third-party narratives.	[70,73,74,105-108,135-137,150-154]
Lack of validated measures specific to post-ABI sexual dysregulation	Develop instruments capturing observable behaviors, setting, redirectability, insight, consent concerns, distress, caregiver impact, safety actions, and appropriate intimacy goals.	Supports standardized phenotyping and treatment evaluation.	Measures should capture dignity, relational goals, and safe intimacy, not only incident reduction.	[139-149,179,186]
Ethical and forensic complexity	Distinguish consent to research from clinical care; provide communication support, authorized representation when applicable, attention to assent/dissent, and reassessment when capacity changes. Embed privacy and safeguarding procedures in the approved protocol.	Improves responsible research conduct and clinical translation.	Refusal of optional intimate questions must not compromise care. Collect only necessary data and justify any recording technology; research consent is not consent to sexual activity or unrestricted data reuse.	[156,157,187-190,196-200,216]
Limited intervention evidence	Grade direct post-ABI intervention evidence by study design and directness; separate environmental, behavioral, psychological, pharmacological, adverse-effect, and safeguarding outcomes.	Makes weak direct evidence and non-ABI extrapolation explicit and supports proportionate clinical claims.	OCEBM levels aid retrieval and appraisal but do not by themselves constitute recommendations; consent, monitoring, and ethical safeguards remain necessary.	[21,22,32,33,154,157,159,160,162-175,183-185,213,214]
Insufficient neurorehabilitative translation	Integrate neuropsychology, psychiatry, nursing, rehabilitation therapy, caregiver support, sexual health, and ethics into shared formulations.	Links mechanisms to individualized care pathways.	Risk management should not become blanket suppression of sexuality or partnership goals.	[11,12,21,22,70,105,107,150]
Sex and gender bias	Report sex assigned at birth and gender identity when relevant and consented; stratify outcomes where feasible; record caregiver/observer gender and relationship role.	Clarifies underrepresentation, gendered reporting thresholds, and possible clinical or forensic selection bias.	Avoid treating sex and gender as interchangeable or inferring sex-specific effects from sparse cases.	[5,6,17-20,27,60-62,95,96,104-108]

Abbreviations: ABI, acquired brain injury; TBI, traumatic brain injury. **Panel B is a proposed documentation aid, not a validated instrument or diagnostic algorithm.**

Panel B Proposed minimum behavioral observation log

Use a shared protocol and neutral definitions. These fields are a proposed documentation aid, not a validated scale, a diagnostic cutoff, or a legal capacity assessment. Clinical records and research extracts require different access controls; record only information necessary for the stated purpose.

Field	Minimum recording rule
**Observation interval and opportunity**	Define the observation period, setting, and relevant opportunity. Distinguish an adequately observed interval without the target behavior from an unobserved interval; record changes in supervision or privacy.
**Source and reliability**	Identify patient self-report, caregiver report, or direct staff observation and the observer role. Separate time of occurrence from time of report; note communication or recall limitations.
**Subjective function and desire**	Record the patient's account of desire, arousal/function difficulties, distress, and intimacy goals separately from observed acts; do not infer increased libido from an incident count.
**Antecedent and context**	Describe the immediate trigger, personal-care task, fatigue, unstructured time, interpersonal cue, or privacy problem using only necessary contextual details.
**Observable target behavior**	Use a predefined neutral description; record count, duration, or recurrence when observable. Distinguish speech, intrusive contact, exposure, perseveration, and consensual expression without inferring preference or intent.
**Medical and medication context**	Record relevant confusion, sleep/mood change, pain, seizures, substances, and medication initiation or dose change. Indicate what is known, unreported, or awaiting assessment.
**Insight and redirection**	Record awareness, the cue or alternative activity offered, response, persistence, and recurrence. Describe what happened rather than applying an unvalidated severity score.
**Consent and immediate safety**	Document agreement/refusal, coercion or vulnerability concerns, and necessary immediate protective actions. Refer to a separate decision-specific capacity assessment; the log does not determine legal capacity.
**Intervention and consequence**	Record environmental, behavioral, educational, and pharmacological actions separately, including staff consistency and opportunity changes that may explain apparent improvement.
**Benefit and harm**	Track safety, distress, participation, redirectability, and preserved appropriate intimacy alongside cognition, sedation, falls, or other adverse effects; fewer incidents alone do not establish benefit.
**Review and confidentiality**	Name the responsible clinician and review time, with criteria to continue, modify, reduce, or stop an intervention. Use coded identifiers for research extracts and omit unnecessary identifying details about patients or third parties.

Supplementary Table S8. Evidence identified through the final 6 August 2026 search and contribution to the review.

This table distinguishes direct sexual-health or implementation evidence from domain-general mechanistic evidence added in the final 6 August 2026 search. Mechanistic studies are not treated as direct validation of post-ABI sexual dysregulation.

Study and design	Sample or scope	Directness to post-ABI sexual dysregulation	Contribution to this review	Principal limitation
Ozkan et al., 2025; systematic review and meta-analysis [13]	279 prospective stroke cohorts; 117,440 participants	Indirect for dysregulation; direct for patient-reported nonmotor outcomes	Establishes that sexual dysfunction after stroke is common and should not be conflated with rare dysregulation	High heterogeneity; hypersexuality and disinhibition were not quantified
Patsakos et al., 2024; clinical guideline [21]	Twelve TBI sexuality recommendations	Direct for sexuality-care organization; mostly indirect for dysregulation treatment	Supports early education, individualized assessment, written resources, relationship coaching, and trained services	Two Level A, one Level B, and nine Level C recommendations; not an efficacy trial
Hwang et al., 2026; mixed-method co-design and implementation study [22]	81 TBI participants, 26 clinician surveys, and 15 interviews	Direct for rehabilitation-service implementation; indirect for dysregulation treatment efficacy	Shows feasible improvement in sexuality support, help-seeking, and clinician attitudes	Single service; continued need for champions; no specific hypersexuality/disinhibition efficacy outcome
Aljboor et al., 2024; systematic review and meta-analysis [65]	52 TBI studies; 7,367 participants	Direct for post-TBI pituitary dysfunction; indirect for sexual dysregulation	Supports endocrine screening when fatigue or reduced desire is clinically compatible	Endocrine dysfunction more plausibly explains reduced function than hypersexuality
Bryant et al., 2023; TRACK-TBI cohort [94]	1,057 participants with TBI	Indirect; cognitive outcomes rather than sexual behavior	Supports heterogeneous cognitive profiles and individualized formulation	No sexual-regulation outcome
Mallas et al., 2025; randomized imaging study [6]	43 TBI participants; imaging sample 28	Indirect mechanistic evidence	Shows treatment-related corticostriatal connectivity and executive-function change	Small imaging sample; no sexual outcome
Kashou et al., 2024; systematic review [98]	50 resting-state fMRI studies; 1,397 TBI and 1,179 controls	Indirect mechanistic evidence	Demonstrates methodological and clinical drivers of network heterogeneity	Mixed significant, null, increased, and decreased connectivity results; no sexual outcome
Terneusen et al., 2023; systematic review [64]	ABI self-awareness literature	Indirect mechanistic evidence	Supports frontal, white-matter, cingulate, and inferior-frontal contributions to impaired self-awareness	No sexual outcome and heterogeneous awareness measures
Akdeniz et al., 2025; lesion-network study [112]	565 ischemic-stroke patients	Indirect methodological evidence	Shows that lesion-network maps may reflect symptom topography without improving prognosis beyond clinical variables	No sexual outcome; limited incremental prognostic value

Study and design	Sample or scope	Directness to post-ABI sexual dysregulation	Contribution to this review	Principal limitation
Ríos et al., 2025; two prospective lesion-network cohorts [113]	377 ischemic-stroke patients across two cohorts	Indirect methodological evidence	Demonstrates the need for replication and cautious interpretation of lesion-network associations	Depressive rather than sexual outcome; weak or non-replicated associations

Abbreviations: ABI, acquired brain injury; fMRI, functional magnetic resonance imaging; TBI, traumatic brain injury.
